# Winter cover crops increase readily decomposable soil carbon, but compost drives total soil carbon during eight years of intensive, organic vegetable production in California

**DOI:** 10.1371/journal.pone.0228677

**Published:** 2020-02-06

**Authors:** Kathryn E. White, Eric B. Brennan, Michel A. Cavigelli, Richard F. Smith

**Affiliations:** 1 United States Department of Agriculture, Agricultural Research Service, Sustainable Agricultural Systems Laboratory, Beltsville Agricultural Research Center, Beltsville, Maryland, United States of America; 2 United States Department of Agriculture, Agricultural Research Service, Salinas, California, United States of America; 3 University of California Cooperative Extension, Salinas, California, United States of America; Michigan State University, UNITED STATES

## Abstract

Maintaining soil organic carbon (SOC) in frequently tilled, intensive organic vegetable production systems is a challenge that is not well understood. Compost and cover crops are often used to add organic matter to the soil in these systems. Compost contributes relatively stabilized carbon (C) while cover crops provide readily degradable (labile) organic matter. Our objectives were to quantify C inputs, and to assess the effects of urban yard-waste compost, winter cover crop frequency and cover crop type on SOC and labile C stocks during eight years of intensive, organic production that usually included two vegetable crops per year in a long-term systems study in Salinas, California. Total C inputs from pelleted fertilizer, compost, vegetable transplant potting mix, vegetable residue and cover crops, including estimates of below ground inputs, ranged from 40 to 108 Mg ha^-1^ in the five systems evaluated. Following a rapid decline in SOC stocks in year 1, compost had the largest effect on SOC stocks increasing mean SOC over years 2 to 8 by an average of 9.4 Mg ha^-1^, while increased cover crop frequency (annual vs. quadrennial) led to an additional 3.4 Mg ha^-1^ increase. In contrast, cover cropping frequency had the largest effect on permanganate oxidizable labile C (POX-C), increasing POX-C by 26% after 8 years. Labile POX-C was well correlated with microbial biomass C and nitrogen. Compost had the greatest effect on total SOC stocks, while increasing cover crop frequency altered the composition of SOC by increasing the proportion of labile C. These results suggest that frequent winter cover cropping has a greater potential than compost to increase nutrient availability and vegetable yields in high-input, tillage intensive vegetable systems.

## Introduction

Intensive cool season vegetable cropping systems in California’s Salinas Valley rely on frequent tillage operations to prepare the soil prior to crop planting. The relatively mild Mediterranean-type climate allows growers to produce multiple crops such as lettuce and broccoli each growing season. Typical tillage operations prior to planting include subsoiling, several rounds of disking, landplaning, listing beds, and bed shaping [1]. Tillage increases short-term soil aeration, incorporates fresh organic matter in the form of crop residues and exposes soil organic matter (SOM) that was physically protected prior to tillage to microbial attack, consequently increasing mineralization rates and loss of soil C [2–4]. Low rates of residue return from crops such as lettuce, along with the relatively low carbon (C):nitrogen (N) ratios of vegetable crop residues [5] result in rapid mineralization following incorporation. These factors combined with the aggressive tillage regime make maintaining SOM in vegetable production systems a major challenge. Therefore, over time long-term intensive vegetable production can lead to a decline in soil organic carbon (SOC, a proxy for SOM) stocks, soil biological activity and soil physical properties [6]. In the Salinas Valley region of California, a major center of U.S. vegetable production, decades of cultivation has resulted in large SOC losses [7].

Organic production systems rely on management of SOM via applications of compost, manure-based and other non-synthetic fertilizers and green manures (cover crops) to maintain soil fertility and crop productivity [8,9]. Compost applications and cover crops can help counteract the negative effect of intensive vegetable production on SOC [9–15]. However, because it is well known that soil C changes slowly over time, the long-term effects of management on SOC changes in tillage-intensive vegetable systems are not well understood because few of these studies exceeded 4 years (e.g. Cogger et al. [15]). Because it has already undergone partial decomposition, mature compost contributes relatively stabilized C to soil contributing slowly available SOC, while cover crops contribute to actively cycled SOC pools [11, 16–18]. Compost application increases the proportion of lignin in SOC as compost-derived cellulosic polysaccharides are preferentially degraded in soil [19]. Long-term compost application (20 years) has been positively correlated with increases in SOC, increasing by stocks by 0.198 Mg C ha^-1^ yr^-1^ [20]. In contrast, cover crop residues decompose rapidly following incorporation due to their low C:N ratio. Consequently, while cover crops increase SOC [21], they also contribute to increased particulate organic matter (POM) over the long term [22,23]. For example, a rye cover crop planted after both phases of a corn silage–soybean rotation increased POM at the 0 to 5 cm depth by 2.3 g kg^-1^ soil over 10 years [23]. Consistent with this rapid microbial processing, cover crops (alone or in combination with manure) can increase POM occluded within soil aggregates in organic production systems; which, due to a lower C:N ratio relative conventional systems, represents increased potential mineralizable N reserves for crop uptake [23,24]. However, the consistency of this effect within organic or any other systems likely depends on the organic matter input rate and the frequency of specific management practices such as cover cropping, which can vary greatly even among certified organic systems growing the same crops [25–27].

In the Salinas Valley, urban yard waste compost is commonly applied to intensively managed vegetable production fields to counteract the loss of SOC, while cover crops are less commonly used due to concerns over their impact on the timeliness of field operations under intensive production schedules [28]. Yard waste compost is likely more resistant than cover crop residue to soil microbial attack as it is largely derived from woody, lignified starting materials and thus is less likely to contribute labile SOC and provide substantial nutrients to subsequent cash crops. For example, mature yard waste compost has been shown to increase total C by an average of 133% and the lignin phenolic component of total C by 179% relative to an unamended control soil, while polysaccharide C was only increased by 18% [19]. Relatively little work has been reported on the effects of compost and cover crops in Salinas Valley production systems. Jackson et al. [11] demonstrated increased microbial biomass C (MBC) and N (MBN) as well as increased yields of lettuce and broccoli following a rye cover crop and compost application in a two-year study. The ongoing long-term Salinas Organic Cropping Systems (SOCS) study is the only other study examining the individual and combined effects of cover crops and compost in the high input, high value cropping systems in this region. The experiment began in 2003 to assess the effects of compost application, cover cropping frequency and cover crop type on organic vegetable production [29]. Related work from the project has already demonstrated that decomposition of legume-rye and mustard cover crops increase soil N 30 to 40 days post incorporation [24]. In addition, annual cover cropping increases MBC, MBN and several soil enzymes relative to quadrennial cover cropping when sampled after 6 years, and had a positive effect on the structure and function of the soil food web [18,30,31].

The objectives of this paper are to quantify C inputs and assess the effects of compost, cover crop frequency and cover crop type on SOC and labile C during the first eight years of intensive production using typical regional vegetable production practices. Specific hypotheses are: i) relatively stabilized and resistant C provided by compost has a greater effect on total SOC, while winter cover cropping increases labile C stocks by adding readily degradable C, ii) increasing cover crop frequency (annually vs. quadrennially) increases SOC and labile C stocks relative to quadrennial cover cropping by increasing the rate and quantity of C input to soil, iii) different cover crop types (legume-rye vs. mustard vs. rye alone) have a differential effect on SOC and labile C stocks reflecting differences in biological N fixation in legume-rye, biomass production, and the resulting C inputs to soil.

## Materials and methods

### Field site and management

A detailed description of the field site, it’s history and the SOCS experimental design is given in Brennan and Boyd [29]. The study was initiated in 2003 on certified organic land at the USDA-ARS in Salinas, CA (36°37ʹN, -121°32ʹW). As this study was part of a USDA-ARS internally funded project conducted at a USDA-ARS research facility there were no special permissions required to conduct the research at this site. Furthermore, the field studies did not involve endangered or protected species. The site has been certified organic since 1999 by California Certified Organic Farmers and by the USDA National Organic Program since its inception in 2002. From 1990 to 1996 the field was utilized for hay production and mixed vegetable and sugar beet trials. From 1997 to 2003 the field was used for occasional vegetable trials and cover crops with minimal compost or fertilizer inputs and frequent fallow periods. The soil is a Chualar loamy sand (fine-loamy, mixed, superactive, thermic Typic Argixerol; 77% sand, 15% silt, 8% clay). In the year prior to plot establishment the field was winter cover cropped with a legume-rye mixture (10% ‘Merced’ rye (*Secale cereale* L.), 35% faba bean (*Vicia faba* L.), 25% ‘Magnus’ pea (*Pisum sativum* L.), 15% common vetch (*Vicia sativa* L.) and 15% purple vetch (*Vicia benghalensis* L.). The field was then summer cover cropped with vetch-mustard (95% common vetch, 5% *Brassica juncea* Czern.) followed by buckwheat (*Fagopyrum esculentum* Moench). In addition, in the year prior to the onset of the experiment, the soil was amended with approximately 22 Mg ha^-1^ (wet weight) urban yard waste compost and approximately 12.3 Mg ha^-1^ mined 75% gypsum.

The experimental design is a randomized complete block with eight systems in four blocks. Each system plot is 19.5 m x 12.2 m. The five systems with optimal cover crop seeding rates for weed suppression (Brennan, unpublished data) are included in this paper (Table 1). In this study, optimal seeding rates for weed suppression were considered to be seeding rates that provided rapid ground cover by the cover crop which thus suppressed weeds and prevented them from producing seeds. These are the same systems previously examined for soil phosphorus and microbial community dynamics [18,32,33]. The experiment began with either a winter fallow (Systems 1 and 2) or a winter cover crop planted in October 2003 (Systems 3, 4 and 5). Cover crop treatments consisted of a legume-rye mix (System 3; seeding rate: 420 kg ha^-1^ that by seed weight included 10% ‘Merced’ rye, 35% faba bean, 25% ‘Magnus’ pea, 15% common vetch and 15% purple vetch), a mustard mix (System 4; seeding rate: 11 kg ha^-1^; 61% ‘Ida Gold’ white mustard (*Sinapis alba* L.), 39% ‘Pacific Gold’ India mustard (*Brassica juncea* Czern.), or ‘Merced’ rye alone (System 5; seeding rate: 90 kg ha^-1^). During the eight years examined Systems 1 and 2 were cover cropped with legume-rye in years 4 and 8 and were left bare fallow in all other winters, while Systems 3, 4 and 5 were cover cropped with their respective cover crop type each winter. Cover crops were planted at 15 cm spacing using a grain drill equipped with cones to facilitate the different cover types at different seeding rates [34].

**Table 1:** The five systems at the Salinas Organic Cropping Systems experiment that were evaluated in this analysis.

System	Total Compost Applied (oven dry basis)	Cover Crop	
	(Mg ha^-1^)	Type	Frequency
1	0	Legume-rye	Quadrennial
2	114	Legume-rye	Quadrennial
3	114	Legume-rye	Annual
4	114	Mustard	Annual
5	114	Rye	Annual

In February or March of each year plots in all five systems were tilled with a soil spader to incorporate the cover crops and prepare the soil for bed formation; thus, the same level of primary tillage occurred in plots with cover crops present annually (Systems 3 to 5), and in those that were fallow most winters (Systems 1 and 2). Following tillage, cover crops were left to decompose for 30 to 42 days, except for year 6 when the period was 72 days due to wet spring weather. Lister plows were used to form 101.6 cm wide peaked beds. Systems 2, 3, 4 and 5 received 7.6 Mg ha^-1^ (dry weight basis) urban yard waste compost (Z-Best Products, Gilroy, CA) while pelleted poultry manure/feather meal based organic fertilizer (Foster Poultry Farms 4-4-2, Livingston, CA; True Organic 8-1-1, Helm, CA) at a rate of 55–66 kg nitrogen (N) ha^-1^) was applied in all systems. The compost was only analyzed in 4 of the 8 years and had a C:N ratio that ranged from 18 to 28 with a mean of 22. The compost was made from a mixture of green and brown trimmings from urban yards in the region where the study occurred. Beds were top-shaped to incorporate the compost, creating approximately 50 cm flat bed tops. The annual cropping sequence for all five systems included transplanted romaine lettuce (*Lactuca sativa* L. var. *longifolia* Lam.) from May to June, which received liquid organic fertilizer (Biolizer GP 2.5-2-1.5, California Liquid Fertilizer, Gonzales, CA; Agrolizer 6-2-0, AgroMar, San Diego, CA; Tierra Fertil 5-1-1, Mar Y Tierra Fertilizantes Orgánicos, Ensanada, Mexico) via drip irrigation to bring the total N application for each crop of lettuce to 73 kg ha^-1^. After the lettuce harvest, beds were either reshaped with a reduced-tillage disk or flattened with a tandem disk and spader and reformed, depending on field conditions. An additional 7.6 kg ha^-1^ of yard waste compost was also applied to Systems 2, 3, 4 and 5 when preparing the beds for the second vegetable crop, baby leaf spinach (year 1, July–August 2004) or transplanted broccoli (all other years, July–September/October). Broccoli was not grown following lettuce during year 8 because the field was transitioned to a year of strawberry production at that time. Spinach received 22 kg N ha^-1^ in pelleted organic fertilizer, while broccoli received pelleted and liquid fertilizers (via drip irrigation) totaling 134–170 kg N ha^-1^. Following harvest of the summer vegetables, plots were subsoiled to approximately 1 m using ripper shanks to ameliorate compaction caused by the heavy equipment used for commercial harvest operations. Crops were harvested and marketed by collaborating farms, except for the 2004 spinach crop and the 2005 broccoli crop which did not meet market standards. These crops and all harvest residues in other years were incorporated by disking, spring-tooth harrowing, soil spading and ring rolling prior to the winter fallow or cover crop treatments.

### Carbon inputs

For each plot, cover crop shoot C inputs were calculated based on previously published biomass production data [29], and season-end cover crop biomass C concentrations of 441 g C kg^-1^ (legume-rye), 421 g C kg^-1^ (mustard) and 442 g C kg^-1^ (rye) [24] from the same study measured by combustion analysis. Biomass was sampled from one 50 by 100 cm quadrat oriented to include three adjacent rows from each plot prior to cover crop incorporation. Samples were oven dried at 65 °C for at least 48 hours until the weight had stabilized and ground (0.250 mm) for total C analysis by the University of California-Davis Analytical Laboratory following additional sample drying at 105 °C (https://anlab.ucdavis.edu/analysis/Plant/522) using a TruSpec CN analyzer (LECO Corp., Saint Joseph, MI). Vegetable post-harvest residues were estimated based on measured harvested shoot biomass and measured harvest indices of 0.26 and 0.24 for romaine lettuce hearts and broccoli, respectively (Brennan, unpublished data). Thus, approximately 74% (1–0.26) of total lettuce shoot biomass and 76% (1–0.24) of total broccoli shoot biomass was left in the field as residue. Vegetable shoot residue C was calculated assuming a shoot C concentration 368 kg C ha ^-1^ [5]. Total C input in fertilizers, compost and transplant potting mix was calculated from the measured cumulative mass of each material used and a C concentration of 280 g C kg^-1^ (4-4-2), 370 g C kg^-1^ (8-1-1), 317 g C kg^-1^ (compost) and 137 g C kg^-1^ (potting mix). Total C concentrations were determined by combustion analysis of ground (0.250 mm) and dried (105 °C) samples using a using a TruSpec CN analyzer (LECO Corp., Saint Joseph, MI). Total organic matter inputs for each of the above categories is shown in the Supporting Information (S1 Fig). We estimated below ground C inputs from cover crop and vegetable roots and root exudates based on above ground biomass, estimates of root exudates, and shoot:root ratios for the cover crop components and the vegetables under field conditions in the literature [35–39], and assuming that the C concentrations of the above and belowground biomass were the same. Specifically, to estimate the root biomass from our measurements of shoot biomass we assumed the following shoot:root ratios of 5.6 for rye [37, 39], 4.5 for legumes [36], 6.3 for mustard [37], 4.1 for lettuce [38] and 4.7 for broccoli [37, 39]. The rye and mustard shoot:root ratios were averages from winter cover crops at the end of the typical cover cropping period (March) in Salinas, CA when these cover crops are typically in the early flowering stage. Shoot:root ratios for the legume components (vetches, faba bean, pea) were based the mean of reported values for chick pea, lentil and field pea [36]. Vegetable crop shoot:root ratios were taken from published reports of studies conducted under similar conditions in the Salinas Valley [38,39]. Root biomass was calculated by dividing each cover crop component, mustard and vegetable shoot biomass by the appropriate shoot:root. Legume-rye root biomass is the sum of the root biomass each component. Root exudate C was estimated from both cover crop and vegetable root biomass C by multiplying the latter by 0.65 as proposed by Bolinder et al. [35].

We should note that in addition to the C from the pelleted organic fertilizer, a small amount of C was also contributed by liquid fish processing waste-based fertilizers. Starting in year 2 the liquid fertilizer was used to supply less than 25% of the fertilizer N applied to vegetables in all systems. Though we did not measure C in the liquid fertilizer, a concentration of 0.066% C has been reported in the literature [40], suggesting that liquid fertilizers contributed only a small amount of C in this study. Therefore, we did not include this source in the sum of fertilizer C input.

### Soil sampling and carbon analysis

Composite soil samples composed of 20 subsamples collected from the 0 to 30 cm depth were collected from each plot in the fall prior to cover crop planting or winter fallow. Total soil C was determined on all air-dried ground (<0.5 mm) soil samples by the College of Agriculture and Natural Resources Analytical Laboratory at the University of California Davis via combustion (https://anlab.ucdavis.edu/analysis/Soils/320) and inorganic soil C by titration of carbonate and bicarbonate with 0.025N H_2_SO_4_ in a saturated paste extract (https://anlab.ucdavis.edu/analysis/Soils/220). Soil organic C was calculated as the difference between total and inorganic soil C. Soil bulk density measured on the top of the broccoli beds in intact samples collected between 2 vegetable transplants from 4 separate beds per plot to a depth of 12.8 cm at the end of years 3 and 7 in each system (Brennan, unpublished data) was used to convert SOC concentrations to SOC stocks using the maximum equivalent soil mass method [41]. This method corrects for errors in SOC stock measurements to a fixed depth due to changes in soil bulk density over time and allows comparisons based on the same soil mass per unit land area. Ideally, corrections are based on the original bulk density at the start of the study; however, because we did not measure bulk density in year 0 and our data showed that bulk density in Systems 2, 3, 4 and 5 decreased during the study (Brennan, unpublished data), the maximum equivalent soil mass method uses the greatest bulk density measured during the study as a proxy for this measurement. Given the intensity of tillage by soil spading to 30 cm, calculations were made with assumption that bulk density was uniform throughout the 0 to 30 cm depth.

Additional soil samples were collected 13 October 2003 (time zero) and 30 October 2009 (year 6) and frozen (-25 °C) for soil microbial biomass analyses from the study that were published previously [18] and based on the chloroform fumigation-extraction method. Briefly, duplicate 15 g subsamples of field moist equivalent soil were fumigated for 24 hr at ambient temperature. The C and N in fumigated and non-fumigated soil samples were extracted with 0.5 M K_2_SO_4_ and analyzed using a Shimadzu TOC/VCPH-TN C/N analyzer (Shimadzu, Kyoto, Japan). Results from non-fumigated samples were subtracted from fumigated samples, and K_EC_ = 0.45 and K_EN_ = 0.54 were used [18]. Samples in 2003 consisted of six composited cores (6.5 cm diameter x 6.7 cm depth), while in 2009 eight cores of this dimension were composited and kept frozen at -25 °C. Thawed subsamples of this archived soil were air-dried and ground (<2 mm) for an additional permanganate oxidizable C (POX-C) analysis [42,43]. In addition, POX-C was measured in soil samples collected from 0 to 30 cm in August of year 8 following lettuce harvest and bed disking (June) and ripping and spading (August). Determination of POX-C was carried out on 2.5 g of soil shaken with 10 ml of a 0.02M KMnO_4_-0.1M CaCl_2_ solution in 50 ml centrifuge tubes for 2 min. Following shaking, the tubes were left for 10 min to allow the soil to settle. A 0.5 ml aliquot of the supernatant was diluted with H_2_O to 50 ml and absorbance measured at 550 nm using a Molecular Designs (San Jose, CA) VersaMax microplate reader. The concentration of KMnO_4_ remaining after reaction with the soil was determined by comparison with a standard curve prepared from dilutions of the stock reagent. The quantity of C oxidized was determined assuming stoichiometric oxidation of 0.75 mol C per mol KMnO_4_. As with SOC, POX-C concentrations were converted to stocks to allow for comparisons over time based on the same soil mass per unit land area given changing soil bulk density over time. In addition, we have provided the POX-C concentrations in the Supplemental Information (S2 Fig) to allow for comparison of these results with studies that report POX-C on that basis.

### Statistical analysis

Statistical analyses were conducted using SAS ver. 9.4 (SAS Inst. Cary, NC) and the Exploratory Software for Confidence Interval (ESCI) [44]. To help ourselves and the readers to see the variability, scatter and skewness of the data we present the raw data along with means and 95% confidence intervals (CI) for the 5 systems as suggested by Drummond and Vowler [45], and then use the CIs of the mean difference between pairs of systems to evaluate the effect size of the experimental factors (compost, cover crop frequency, and cover crop type). This approach is consistent with the data presentation used in related published work from the SOCS experiment detailing these treatment effects on MBC, MBN [18] and soil enzyme activity [32], as well as on legume-rye cover crop performance [28]. In addition, S1 Table in the Supplemental Information lists the means, 95% confidence limits and standard errors for all data presented in the figures. For example, to evaluate the compost effect, the CI of the difference between Systems 1 and 2 was used, whereas for the effect of cover cropping frequency the CI of the difference between Systems 2 and 3 was used. The CIs of the paired differences were calculated in ESCI along with a standardized effect size measure (Cohen’s unbiased *d*, hereafter “*d*_unb_”). Cohen’s *d*_unb_ was calculated by dividing the effect size in original units by a standardizer that is based on the standard deviations of the paired two measurements and multiplied by an adjustment factor [46]. This standardized effect size can be used to compare effect sizes regardless of differences in the scale of the units of measurement.

Using this ‘inference by eye’ approach [46,47], the CI of a paired difference that does not include zero provides more evidence of a true effect (P<0.05 comparison-wise error rate) than a CI of a difference that includes zero; where one limit of a difference CI just touches zero, P = 0.05. Furthermore, a wider CI for a difference indicates more uncertainty in the interval estimate. We mention the relationship between CI and P-values merely as a point of reference, but discourage readers from using CIs in a rigid, dichotomous way (i.e., if the CI of a difference includes zero there is no effect). Brennan and Acosta-Martinez [32] provide a more detailed discussion of this inference approach that has been used effectively for analysis of other aspects of the data in this long-term experiment.

We chose to use this approach to statistical inference due to valid criticisms of the reliance on null-hypothesis statistical testing [46–53]. However, we recognize that readers may not be familiar with this approach, therefore we also present the results of a more traditional statistical analysis. To this end, we conducted a repeated measures ANOVA using the MIXED procedure fitted with a first order autoregressive covariance structure using SAS with system, year, and system x year as fixed effects and block as a random effect for analysis of SOC stock for years 2 through 8 as the response variable. Separate ANOVAs were conducted for POX-C stock measured at years 0 and 6 as a repeated measures fitted with a first order autoregressive covariance structure with system, year, and system x year as fixed effects and block as a random effect, and in year 8 using system as the fixed effect and block as a random effect. For year 8, we square root transformed the data to account for variance heterogeneity and present the back-transformed data. We also conducted ANOVAs with the sum of C inputs from cover crop shoots, vegetable shoot residues and total C inputs over 8 years as response variables with system as a fixed effect and block as a random effect. Variances were grouped to model heterogenous variances among the systems. Table 2 lists the *F* statistics and associated level of significance for all ANOVAs. Means separations for all ANOVAs were performed by Tukey-Kramer test (P≤0.05), which controls for family-wise error. We present the results of these analyses with lower case letters placed just above the x-axis so the presentation doesn’t obstruct the ‘inference by eye’ approach [46, 47] discussed above. Our goal is to provide the reader with multiple tools to assess the experimental results, interpretation and conclusions. However, in the text we focus on the data and the magnitude of treatment effects on total SOC stocks and labile C (POX-C), rather than the results of null hypothesis significance testing.

**Table 2:** Carbon inputs, soil organic carbon and permanganate oxidizable carbon ANOVA *F*-statistics and significance.

Effect	Total C Inputs	Cover Crop Shoot C Inputs	Vegetable Residue Shoot C Inputs	Soil Organic C (Years 2 to 8)	Permanganate Oxidizable C (0 to 6.7 cm)	Permanganate Oxidizable C (0 to 30 cm)
System	2572***	788.4***	13.4***	26.7***	21.2***	17.7***
Year				35.7***	308.5***	
System*Year				50.5 n.s.	19.6***	

*** P<0.001

## Results and discussion

### Organic carbon inputs

**Fig 1:**
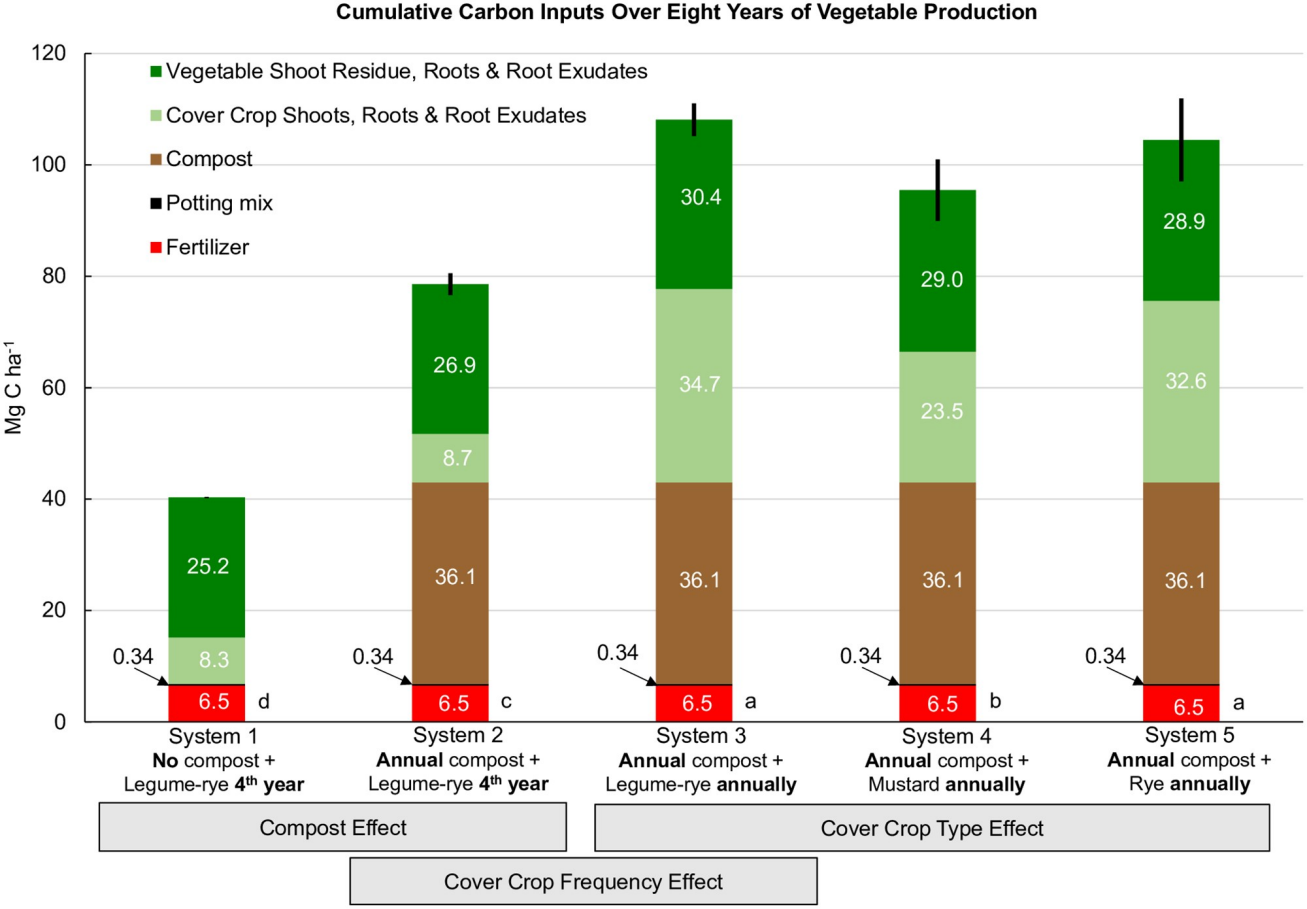
Cumulative carbon inputs over eight years of vegetable production. Sum of mean carbon inputs over eight years of intensive vegetable cropping from fertilizer, vegetable transplant potting mix, urban yard waste compost, cover crops and vegetables in five organic vegetable systems in Salinas, CA. Systems differed by annual compost additions (0 vs. 7.6 Mg ha^-1^ before each vegetable crop), cover crop type (legume-rye, mustard or cereal rye alone) and cover cropping frequency (quadrennially vs. annually planted). Inputs from cover crops include shoots, estimates of roots and root exudates, and for vegetables included post-harvest vegetable shoot residues of broccoli and lettuce and estimates of their roots and root exudates. Bar heights equal the sum of all carbon inputs with the 95% confidence interval error bar for total carbon input. Mean total estimated carbon inputs (Mg ha^-1^) were 40.3 (System 1), 78.5 (System 2), 108.1 (System 3), 95.4 (System 4) and 104.4 (System 5); due to rounding, the sum of the values within the bars for each system may differ slightly from the mean totals. Different lower case letters above the x-axis to the right of each bar indicate that the total carbon means are significantly different based on the Tukey-Kramer adjusted family-wise error rate of (P≤0.05).

**Fig 2:**
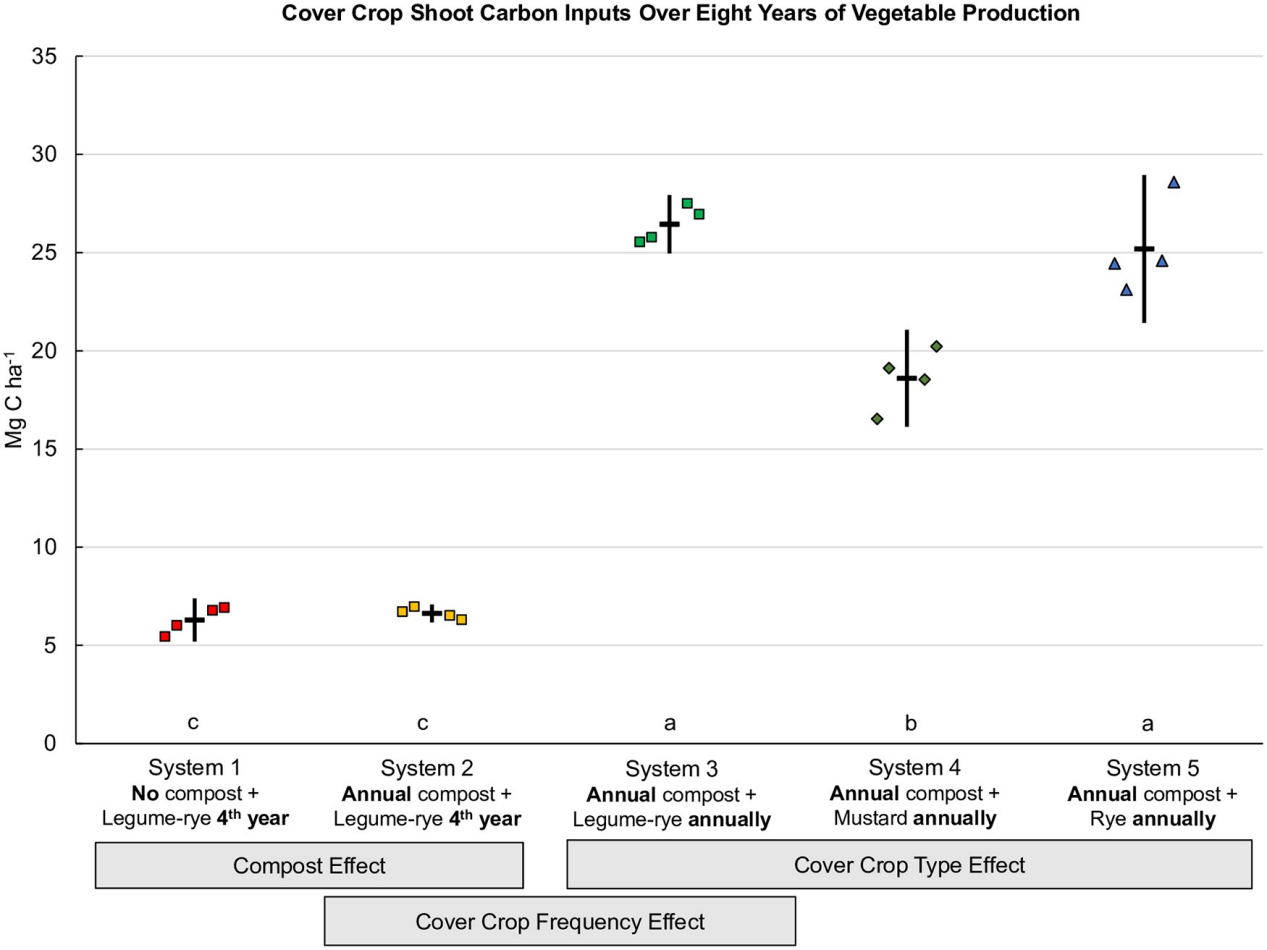
Cover crop shoot carbon inputs over eight years of vegetable production. Measurements were taken in five organic vegetable systems in Salinas, CA. Systems differed by annual compost additions (0 vs. 7.6 Mg ha^-1^ before each vegetable crop), cover crop type (legume-rye, mustard, or cereal rye alone) and cover cropping frequency (quadrennially vs. annually planted). Error bars are 95% confidence limits. Individual data points for reps 1 through 4 of each system are clustered in order from left to right around the mean, which is represented by the horizontal lines. Different lower case letters above the x-axis indicate that the shoot carbon means are significantly different based on the Tukey-Kramer adjusted family-wise error rate of (P≤0.05). Mean cover crop shoot carbon inputs (Mg ha^-1^) were 6.3 (System 1), 6.6 (System 2), 26.4 (System 3), 18.6 (System 4) and 25.2 (System 5).

**Fig 3:**
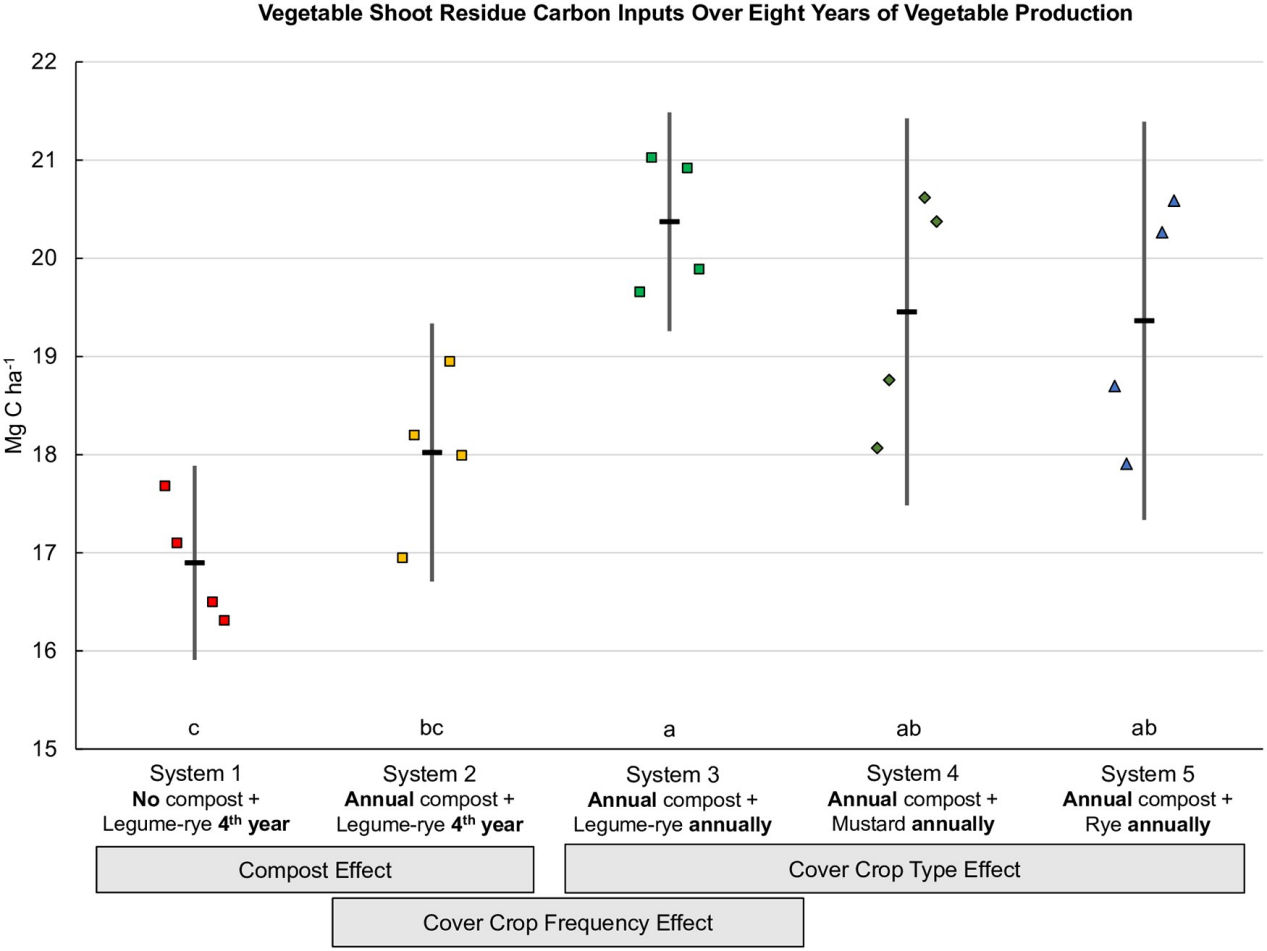
Vegetable shoot residue carbon inputs over eight years of vegetable production. Measurements were taken in five organic vegetable systems in Salinas, CA. Systems differed by annual compost additions (0 vs. 7.6 Mg ha^-1^ before each vegetable crop), cover crop type (legume-rye, mustard, or cereal rye alone) and cover cropping frequency (quadrennially vs. annually planted). Error bars are 95% confidence limits. Individual data points for reps 1 through 4 of each system are clustered in order from left to right around the mean, which is represented by the horizontal lines. Different lower case letters above the x-axis indicate that the shoot carbon means are significantly different based on the Tukey-Kramer adjusted family-wise error rate of (P≤0.05). Mean vegetable residue shoot carbon inputs (Mg ha^-1^) were 16.9 (System 1), 18.0 (System 2), 20.4 (System 3), 19.5 (System 4) and 19.4 (System 5).

Compost was the largest single C input in Systems 2, 3, 4 and 5 (Fig 1), providing a total of 36.1 Mg C ha^-1^ over eight years and accounting for almost all of the 38.2 Mg C ha^-1^ difference in combined C inputs between Systems 1 and 2. As Systems 1 and 2 were only cover cropped twice with the legume-rye mixture during the experiment (between years 3 and 4, and years 7 and 8) (Table 1) cover crop C inputs were similar between the two systems (Fig 2 [28]). Planting the legume-rye cover crop annually as opposed to quadrennially (System 3 vs. System 2) resulted in a 294% increase in cover crop shoot C inputs (Fig 2) and a 11% increase in vegetable shoot residue C returned to the soil relative to System 2 (Fig 3). This increased cover cropping frequency in System 3 resulted in a 90% increase in the amount of on-farm produced C inputs from cover crop shoots and vegetable shoot residues alone, than occurred in System 2 that was bare most winters. The combined C inputs to the soil to System 3 from cover crop shoots and vegetable shoot residues (46.8 Mg ha^-1^, Figs 2 and 3) slightly exceeded the C inputs from off-farm sources (42.9 Mg ha, compost + pelleted organic fertilizers + transplant potting mix, Fig 1). Cover crop shoot C concentrations and biomass yield were similar between the legume-rye and rye cover crops [24,29] and consequently cover crop shoot C inputs were similar between Systems 3 and 5 (Fig 2). In contrast, the mustard cover crop C inputs in System 4 were 30% lower on average than the legume-rye or rye cover crops due to greater cover crop shoot biomass produced in Systems 3 and 5 [29] and the lower C concentration in mustard residues (421 g kg^-1^) than in legume-rye (441 g kg^-1^) and rye (442 g kg^-1^) [24]. Overall, reduced cover crop biomass production resulted in 10.8 Mg ha^-1^ lower C inputs over the 8 years in System 4 relative to mean total C inputs for Systems 3 and 5 (Fig 1). Despite differences in cover crop and vegetable C inputs among cover crop types (System 3, 4 and 5), increasing cover cropping frequency resulted in 16.9 to 29.6 Mg ha^-1^ greater total C inputs to the soil compared to System 2.

Compost and cover cropping frequency had clear effects vegetable biomass production ([54], Brennan, unpublished data) and consequently on C inputs to the soil from post-harvest vegetable residues (Fig 3). For example, compared to System 1, C inputs from the vegetable shoots alone were approximately 1.1 Mg ha^-1^ greater with compost additions (System 2), and an additional 2.4 Mg ha^-1^ with annual cover cropping (System 3). The positive effect of cover crops on C inputs to the soil from vegetables was likely because the cover crop increased nutrient cycling from the vegetables from one year to the next.

**Fig 4:**
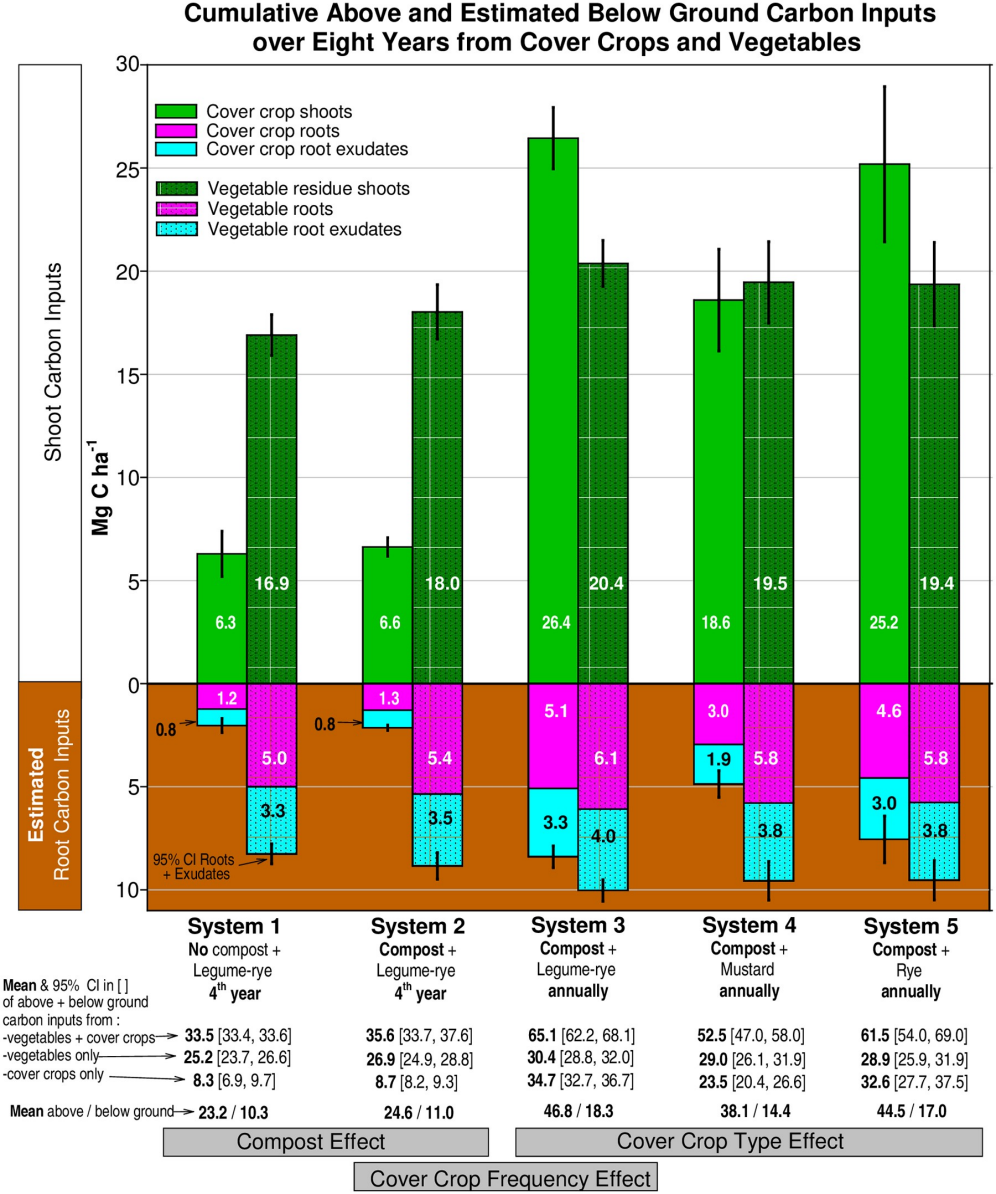
Cumulative above and below ground carbon inputs over eight years from cover crops and vegetables and their estimated below ground inputs from roots and root exudates during 8 years in five organic vegetable systems in Salinas, CA. Systems differed by annual compost additions (0 vs. 7.6 Mg ha-1 before each vegetable crop), cover crop type (legume-rye, mustard or cereal rye alone) and cover cropping frequency (quadrennially vs. annually planted). The grey boxes indicate the systems to compare to evaluate the effects of compost, cover crop frequency, and cover crop type. Error bars are 95% confidence intervals (CI) for shoots, and roots + root exudates. Due to rounding, the sum of the values within the bars for each system may differ slightly from the mean value shown below each system. The assumptions used to estimate the below ground inputs are described in the Materials and Methods.

We estimated that cover crop shoot biomass accounted for approximately 77% of total cover crop C input, whereas C from root biomass and root exudate were approximately 14 and 9%, respectively, and root exudates were approximately 40% of belowground C inputs over 8 years (Fig 4). Increasing cover cropping frequency (System 2 vs. System 3) increased cover crop root C and root exudate C by 292% and vegetable root C and root exudate C by 13%. In all systems, below ground C inputs from vegetables were greater than those from cover crops with the greatest differences with infrequent cover cropping (System 1 and 2) or mustard cover crop (System 3). The effects of compost, cover cropping frequency and cover crop type on the combined above and belowground C inputs from cover crops and vegetables (Fig 4) was similar to those observed for cover crop shoot C inputs (Fig 2).

In sandy loam soils in Georgia, legume cover crops improved yields of silage corn, tomatoes and eggplant by increasing soil N and improving soil physical properties [10]. Jackson et al. [11] reported that a compost application at 9 Mg ha^-1^ (wet weight, ≈ 5.4 Mg ha^-1^ dry weight assuming that the wet compost was 40% moisture) prior to lettuce and broccoli, combined with an annually planted rye cover crop, likewise increased lettuce and broccoli biomass relative to a no compost and no cover crop treatment in a conventional but otherwise similarly managed vegetable production system in the Salinas Valley. Though not reported, these yield increases presumably increased the rate of residue C return to the soil.

### Soil organic carbon

**Fig 5:**
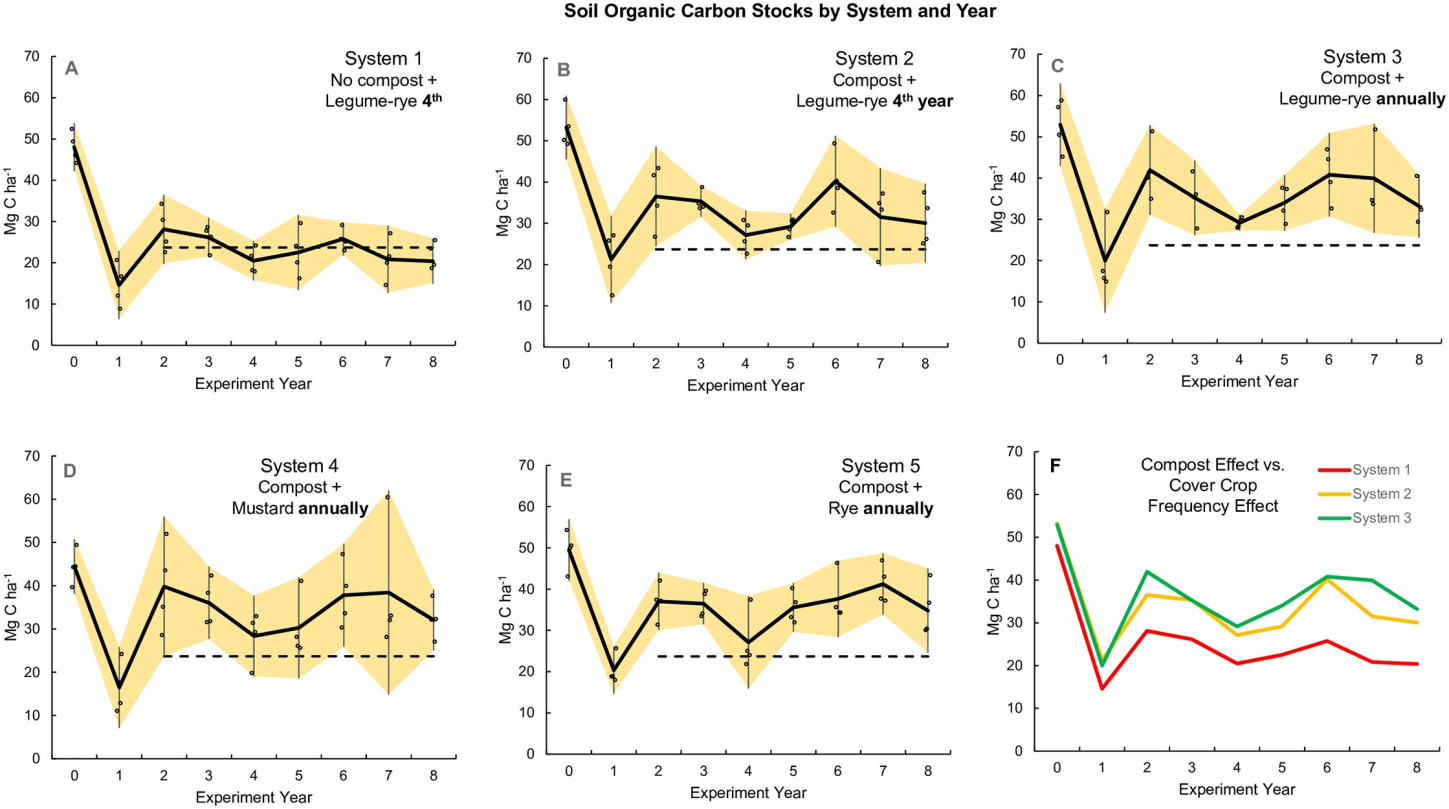
Soil organic carbon stocks by system and year. Measurements were taken at the 0 to 30 cm depth over 8 years in five organic vegetable systems in Salinas, CA. Systems differed by annual compost additions (0 vs. 7.6 Mg ha^-1^ before each vegetable crop), cover crop type (legume-rye, mustard, or cereal rye alone) and cover cropping frequency (quadrennially vs. annually planted). The dashed line indicates mean soil organic carbon between years 2 to 8 for System 1 and is included on each graph as a reference. Error bars are 95% confidence limits that are connected from year to year by the yellow band. Individual data points for reps 1 through 4 of each system are clustered around the mean.

Lower intensity management and use of multiple cover crops during years prior to the start of the experiment, application of 22 Mg ha^-1^ (wet weight) of compost and lower tillage intensity (disking vs. spading) the year prior to the start of the experiment resulted in an average of 50 Mg SOC ha^-1^ for all systems at the start of the study (Fig 5). The imposition of intensive tillage, including spading to 30 cm, and two fertilized and irrigated crops during most years resulted in the loss of 31 Mg SOC ha^-1^ during year 1 averaged across all systems. In addition, the soil C:N ratio declined from 9.6 to 6.7 as SOC declined as well (Table 3). Given the variability in Year 1 SOC stock among systems, no effect of year 1 compost applications or cover crops was apparent between years 0 and 1 (Fig 5A–5E). However, comparison of the compost effect vs. the cover crop frequency effect (Fig 5F) reveals that compost application did buffer the loss to a small degree, while cover crops had no effect. First year SOC stock losses in comparable treatments averaged 22% after initiation of moldboard plowing in a long-term study of hairy vetch cover crops in vegetable production following eight years of alfalfa in a Georgia sandy loam soil [10]. Though the magnitude of the decline was smaller than observed in our study (38% decline), tillage and cropping intensity in the Georgia study were also lower and the slightly finer textured soil may have provided better SOC protection from mineralization.

**Table 3:** Mean soil carbon:nitrogen ratios and 95% confidence limits prior to the start of the experiment and after the first year of intensive vegetable production.

System	Year 0	Year 1
	C:N	C:N
1	9.5 [9.3, 9.8]	5.9 [5.0, 6.7]
2	9.8 [9.5, 10.1]	7.5 [6.5, 8.5]
3	9.7 [9.5, 9.9]	6.8 [6.3, 7.3]
4	9.2 [9.0, 9.3]	6.1 [5.5, 6.6]
5	9.7 [9.6, 9.7]	7.4 [7.0, 7.7]
**Mean**	**9.6 [9.4, 9.8]**	**6.7 [6.1, 7.3]**

The magnitude of SOC decline in our study is likely due to multiple factors. As noted above, less intense management in the field prior to initiation of the study resulted in relatively high SOC. In addition, the roots of the multiple cover crops grown at our study site in the year prior to the start of the experiment likely contributed to soil macroaggregate formation [55–57] that protected SOC prior to year 0. This SOC was likely exposed when tillage disrupted the macroaggregates during year 1. Increasing tillage intensity promotes greater rates of SOC mineralization by increasing soil aeration and crushing soil aggregates, exposing protected SOC and transferring it into pools with substantially shortened soil residence times [3,4,58–60]. The coarse soil texture in our study provides little capacity to physically protect SOC, so any aggregate formation would be readily disrupted by increased tillage intensity. Furthermore, the cover crops grown prior to year 0 were incorporated by disking, which likely only affected the 0 to ~15 cm depth. Thus, the 0 to 30 cm soil samples collected at the start of the experiment may have contained SOC that had accumulated at a depth unaffected by previous tillage. This SOC was then exposed to microbial attack by the new, deeper tillage regime. Finally, the loss of SOC between years 0 and 1 may have been due to increased mineralization resulting from the priming effect of freshly incorporated readily degradable organic C sources such as vegetable residues and organic fertilizers. Fontaine et al. [61] demonstrated that addition of a readily mineralizable C source to soil can stimulate mineralization of SOC, and that this elevated mineralization continues after the introduced C is depleted. This effect may help to explain why compost made little contribution to SOC in year 1 compared to subsequent years as soil microorganisms stimulated by increased soil aeration and C availability rapidly degraded both the newly exposed SOC and compost C along with the readily degradable cover crop, vegetable crop residue and fertilizer C.

Soil organic C stocks increased in year 2 in all systems as SOC responded to the new intensive tillage regime, vegetable crop rotation and compost applications (Systems 2, 3, 4 and 5) (Fig 5). The dashed line in the Fig 5 plot for all systems indicates mean SOC between years 2 to 8 for System 1 and is included as a reference. System 1 SOC increased by 10 Mg ha^-1^ in year 2 relative to year 1 reflecting the response to tillage and crop rotation, while compost application to System 2 increased SOC by 18 Mg ha^-1^. Inclusion of cover crops further increased SOC stocks by 23 Mg ha^-1^, 21 Mg ha^-1^, and 19 Mg ha^-1^ for Systems 3, 4 and 5, respectively. Fluctuations in SOC between years 2 and 8 followed a similar pattern in all systems; however, compost application had the primary effect, increasing mean SOC stock in Systems 2, 3, 4 and 5 relative to System 1. In the three annually cover cropped systems, cover crop type did not appear to have an effect on SOC stocks after 8 years as evidenced by the large overlap and similarities in the year 8 CIs in Mg ha^-1^ of the legume-rye ([25.6, 40.8], Fig 5C), mustard ([25.0, 39.1], Fig 5D), and rye ([24.6, 45.0], Fig 5E). This conclusion is further supported by the 95% CIs of the mean difference in SOC stocks at year 8 between the three annually cover cropped systems where the mean differences were relatively close to zero (System 3 vs. 4: [-10.8, 10.7], *d*_*unb*_ = -0.009; System 3 vs. 5: [-12.1, 15.5], *d*_*unb*_ = 0.23; Systems 4 vs. 5 [-12.6, 16.1], *d*_*unb*_ = 0.24). A visual comparison of the effect of cover crop type on SOC over time is shown in the Supporting Information (S3 Fig). Despite the lack of a clear cover crop type effect, it is interesting to note that the lower limit of the CI of C stocks exceeded the dashed reference line for years 2 to 8 with legume-rye (System 3), for all years except year 4 with rye (System 5), but only for years 3, 6 and 8 with mustard (System 4). Furthermore, among the 3 legume-rye systems from years 2 to 8 the average SOC stocks were always higher with compost and were usually higher with frequent cover cropping, although the effect of compost (System 1 vs. 2) was greater than that of frequent cover cropping (System 2 vs. 3) (Fig 5F).

Further evidence for a new equilibrium between SOC stocks and the effects of tillage and cropping system management is illustrated in the similar SOC variability among systems over time. The coefficient of variation (CV ± 95% CI) for mean SOC stock over years 2 through 8 for Systems 1 through 5 were: 13% ± 3; 14% ± 4; 13% ± 4; 13% ± 4; and 12% ± 4, respectively. Within-year SOC variability for each system measured by CV ± 95% CI for years 2 to 8 were also similar among Systems 1, 2, 3, and 5, averaging 15% ± 6. However, incorporation of the mustard cover crop clearly increased within-year SOC variability relative to the other annually planted cover crops. The CV of System 4 SOC stock was 22% ± 8, while the CV for System 3 (legume-rye) and System 5 (rye) were 14% ± 5 and 15% ± 5, respectively. These results are consistent with those reported by Brennan and Boyd [29], who found the CV of year end cover crop biomass yields for Systems 3, 4 and 5 were 13% ± 4, 21% ± 5 and 16% ± 5, respectively.

**Fig 6:**
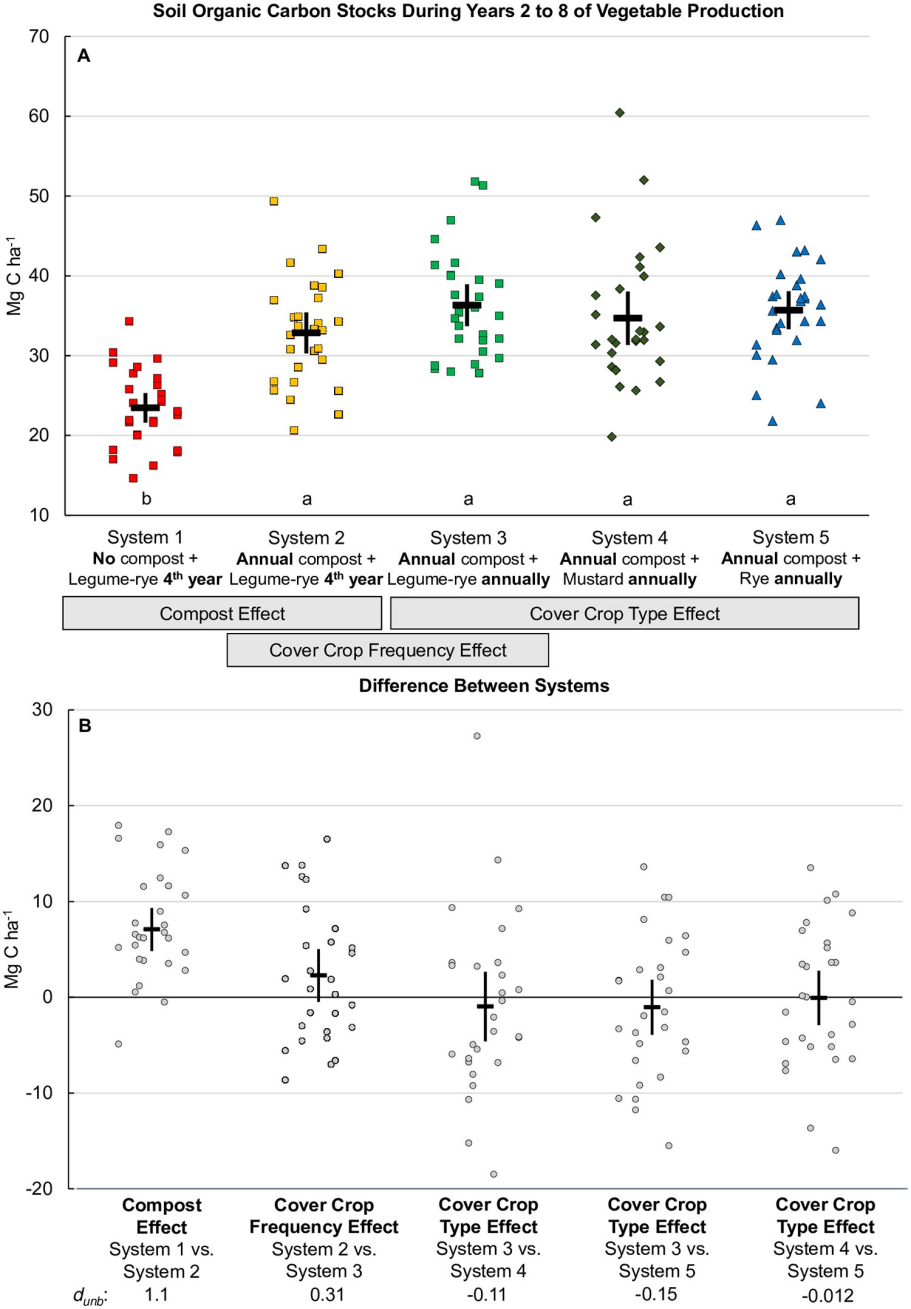
Soil organic carbon stocks during years 2 to 8 of vegetable production. Measurements were taken at the 0 to 30 cm depth. Mean soil organic carbon stocks for years 2 through 8 (A) and mean differences between systems (B) in five organic vegetable systems in Salinas, CA. Error bars are 95% confidence limits. Systems differed by annual compost additions (0 vs. 7.6 Mg ha^-1^ before each vegetable crop), cover crop type (legume-rye, mustard, or cereal rye alone) and cover cropping frequency (quadrennially vs. annually planted). In plot A the individual data points are in order of year 2 to 8 and replicate 1 to 4 from left to right; the same order is used in the difference between system pairs. The error bar in the center of the data cluster is the 95% confidence interval with the mean at the horizontal line. Means with the same letter above the x-axis in plot A are not significantly different based on the Tukey-Kramer adjusted family-wise error rate of (P≤0.05). The standardized effect size (Cohen’s unbiased *d*, *d*_unb_) is shown below the x-axis labels in plot B.

Because SOC levels were relatively stable from years 2 through 8, we present the results for these years separately (Fig 6). Mean SOC stocks for years 2 to 8 clearly reveal the dominant effect of compost relative to cover crop frequency or type in these intensive, organic, cool-season vegetable production systems (Fig 6A). The compost effect on SOC is evident in the CI of the difference [4.9, 9.4] between Systems 1 and 2 (Fig 6B) that had a mean of 7.1 Mg ha^-1^. In contrast, increasing the frequency of the legume-rye cover crop (System 2 vs. System 3) had a smaller mean effect (2.3 Mg ha^-1^) on SOC stock, but the small overlap in the CI of this difference [-0.5, 5.0] with zero and the scatter of nearly half of the raw difference points below zero provides more uncertainty in the effect of cover cropping frequency than of compost; this agrees with the trends in Fig 5F. Finally, differences in cover crop type had no consistent effect on SOC stocks (Fig 6B).

Compost application increases the proportion of lignin and aromatic moieties of SOC, which, because they are resistant to microbial attack, persist relatively unaltered as the remainder of compost C degrades in soil [19]. Given the nature of the feedstocks used in preparing the urban yard waste compost used in our study, it’s likely that compost contributed more resistant C to SOC over time. Long-term application of urban yard waste compost has been shown to increase SOC concentration by 17% in a silt loam soil over a fourteen year period [62]. Unfortunately, the authors do not report bulk density so the effect on SOC stocks cannot be assessed. Because compost applications decrease soil bulk density over time [12,63], these increases in SOC concentrations may not reflect increasing SOC stocks. Evanylo et al. [12] showed that application of yard waste compost at rates comparable to those in our experiment (8.6 to 13.4 Mg ha^-1^ y^-1^, dry basis) over a two-year timeframe to a Virginia silty clay loam soil increased SOC stocks (0 to 15 cm) from approximately 26 Mg ha^-1^ to 31 Mg ha^-1^. In contrast, in a conventional lettuce/broccoli production system on a silt loam soil in the Salinas Valley with similar tillage intensity to our study, a split application of compost totaling 18 Mg ha^-1^ y^-1^ (wet weight basis) containing 30% yard waste (in combination with a rye cover crop) did not increase SOC stocks at 0 to 15 cm over two years [11]. The difference in the results between that study and ours on a sandy soil may be due to several differences, including soil texture, the composition of the compost applied, the shorter duration of the study and the long-term history of that site in continuous commercial vegetable production. In addition to yard waste, the compost applied in the earlier study [11] included more degradable salad packing plant waste and horse manure and had a lower C:N ratio than the compost we used. Consequently, their compost may have contained proportionally less resistant C and was more rapidly mineralized following incorporation.

Like the results of our study, other authors have reported a smaller effect of cover crops compared to organic amendments such as compost or manure on SOC stocks. For example, over 14 years the SOC stock change in an organic manure based cropping system was nearly double that of a legume based organic cropping system, though both increased SOC stocks relative to a conventional system [64]. In California’s Central Valley, after 20 years of organic corn (*Zea mays* L.)/tomato (*Lysopersicum esculentum* Mill.) production inputs from annual faba bean—oat (*Avena sativa* L.) cover crops and poultry litter compost increased SOC 3.95 Mg ha^-1^, while in a conventionally managed cover cropped system SOC did not increase [20]. Increased SOC at depth increments below 15 cm was positively correlated with cumulative compost C inputs, but not cover crop C inputs. Cover crop residues, particularly legumes, are rapidly degraded following soil incorporation [20]. Legume-rye mixtures decompose more slowly than pure legume cover crops, with rates decreasing with increasing proportions of rye in the mix [65]. Consequently, legume-rye cover crop mixtures were more effective than rye or legumes alone at slowing tillage-induced SOC loss over time in cotton and sorghum rotations with various cover crop treatments [66]. In contrast, we did not find any differences in SOC due to cover crop type. Furthermore, incorporating pelleted poultry litter, which is similar to the poultry manure based fertilizers in our study, can increase the rate of cover crop degradation [65]. It is possible that the duration of the current study was insufficient to detect a more pronounced cover crop effect on SOC. In a global meta-analysis, time since the introduction of cover cropping was the most important factor affecting a predicted 0.3 Mg ha^-1^ yr^-1^ SOC stock change [67]. Factors such as tillage, cover crop type (legume vs. non-legume) and climate had no effect. However, to our knowledge this meta-analysis was based primarily on agronomic crops with far less intensive tillage than occurred in our study.

### Permanganate oxidizable carbon

**Fig 7:**
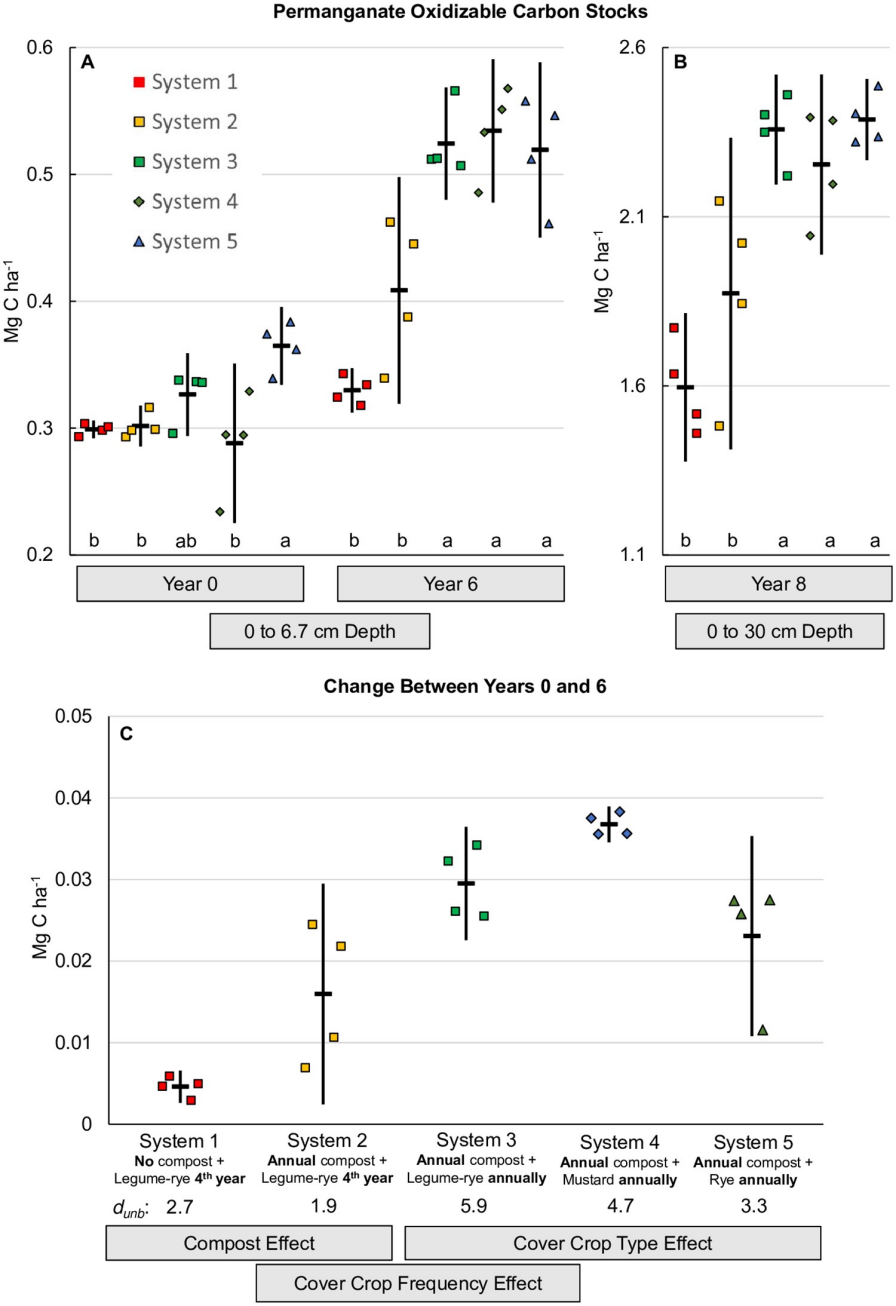
Mean permanganate oxidizable carbon stocks. Measurements were taken at the 0 to 6.7 cm depth for years 0 and 6 (A), the 0 to 30 cm depth in year 8 (B) and the change between year 0 and 6 at the 0 to 6.7 cm depth (C) in five organic vegetable systems in Salinas, CA. Systems differed by annual compost additions (0 vs. 7.6 Mg ha^-1^ before each vegetable crop), cover crop type (legume-rye, mustard, or cereal rye alone) and cover cropping frequency (quadrennially vs. annually planted). Error bars are 95% confidence limits. Individual data points for reps 1 through 4 of each system are clustered in order from left to right around the mean, which is represented by the horizontal lines. Mean permanganate oxidizable carbon stock at the 0 to 6.7 cm depth (Mg ha^-1^) was 24 (System 1), 33 (System 2), 36 (System 3), 35 (System 4) and 36 (System 5). Within a year, means with the same letter above the x-axis in plot A are not significantly different based on the Tukey-Kramer adjusted family-wise error rate of (P≤0.05). The standardized effect size (Cohen’s unbiased *d*, *d*_unb_) is shown below the x-axis labels in plot B.

**Fig 8:**
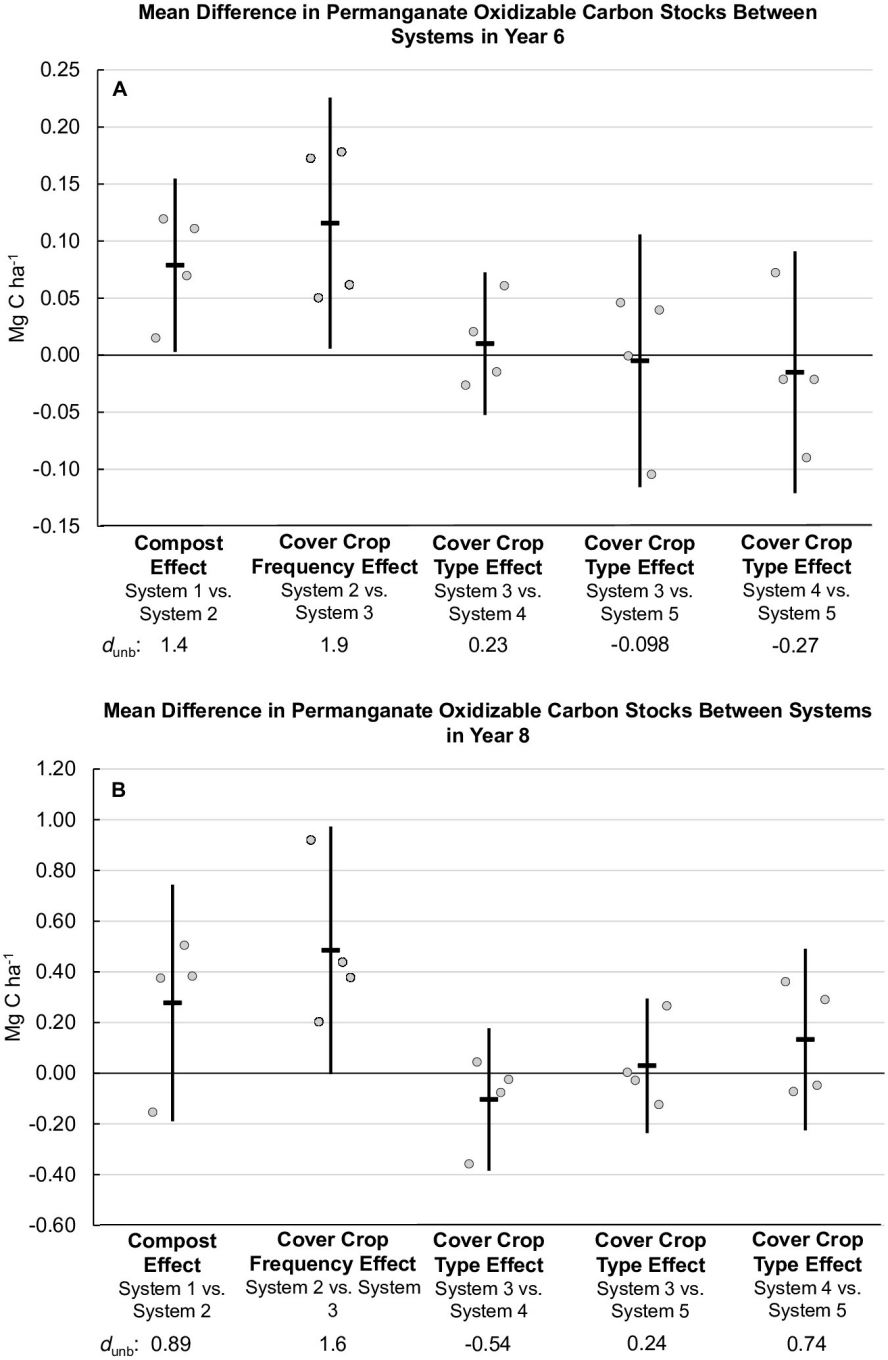
Mean difference in permanganate oxidizable carbon stocks between systems in years 6 and 8. Measurements were taken at the 0 to 6.7 cm depth for years 0 and 6 (A), and the 0 to 30 cm depth in year 8 (B). Systems differed by annual compost additions (0 vs. 7.6 Mg ha^-1^ before each vegetable crop), cover crop type (legume-rye, mustard, or cereal rye alone) and cover cropping frequency (quadrennially vs. annually planted). Error bars are 95% confidence limits. Individual data points for replicates 1 through 4 of each system are clustered in order from left to right around the mean, which is represented by the horizontal lines. The standardized effect size (Cohen’s unbiased *d*, *d*_unb_) is shown below the x-axis labels.

Although the effect of annual cover crops on total SOC was modest (*d*_unb_ = 0.31, Fig 6), the effect on the labile C fraction measured by POX-C (Fig 7) was appreciable (*d*_unb_ = 1.9, year 6, *d*_unb_ = 1.6, year 8, Fig 8), indicating potential benefits of frequent cover cropping on this SOC fraction (Fig 7). Both compost and increased cover crop frequency increased POX-C, while differences in cover crop type did not (Fig 7A). In System 1, six years of minimal C inputs to the soil resulted in an increase in mean POX-C of only 0.005 Mg ha^-1^ (Fig 7C), compared with the 0.016 Mg ha^-1^ increase in System 2 that received compost and the 0.030 Mg ha^-1^ increase in System 3 that received compost and cover crops annually. Six years of annual winter cover cropping with compost increased mean POX-C stocks for legume-rye (System 3), mustard (System 4), and rye (System 5) with the greatest increases for Systems 3 and 4 (Fig 7C), likely due to the lower C:N ratio of the legume-rye and mustard residues relative to rye [24] resulting in greater degradability following incorporation. The mean POX-C stock in Year 6 in System 3 with annual legume-rye cover crops was 59% greater than System 1 and 28% greater than System 2, similar to the mean for all the annually cover cropped systems which were 59% greater than in System 1 and 29% greater than in System 2 (Fig 7A).

**Fig 9:**
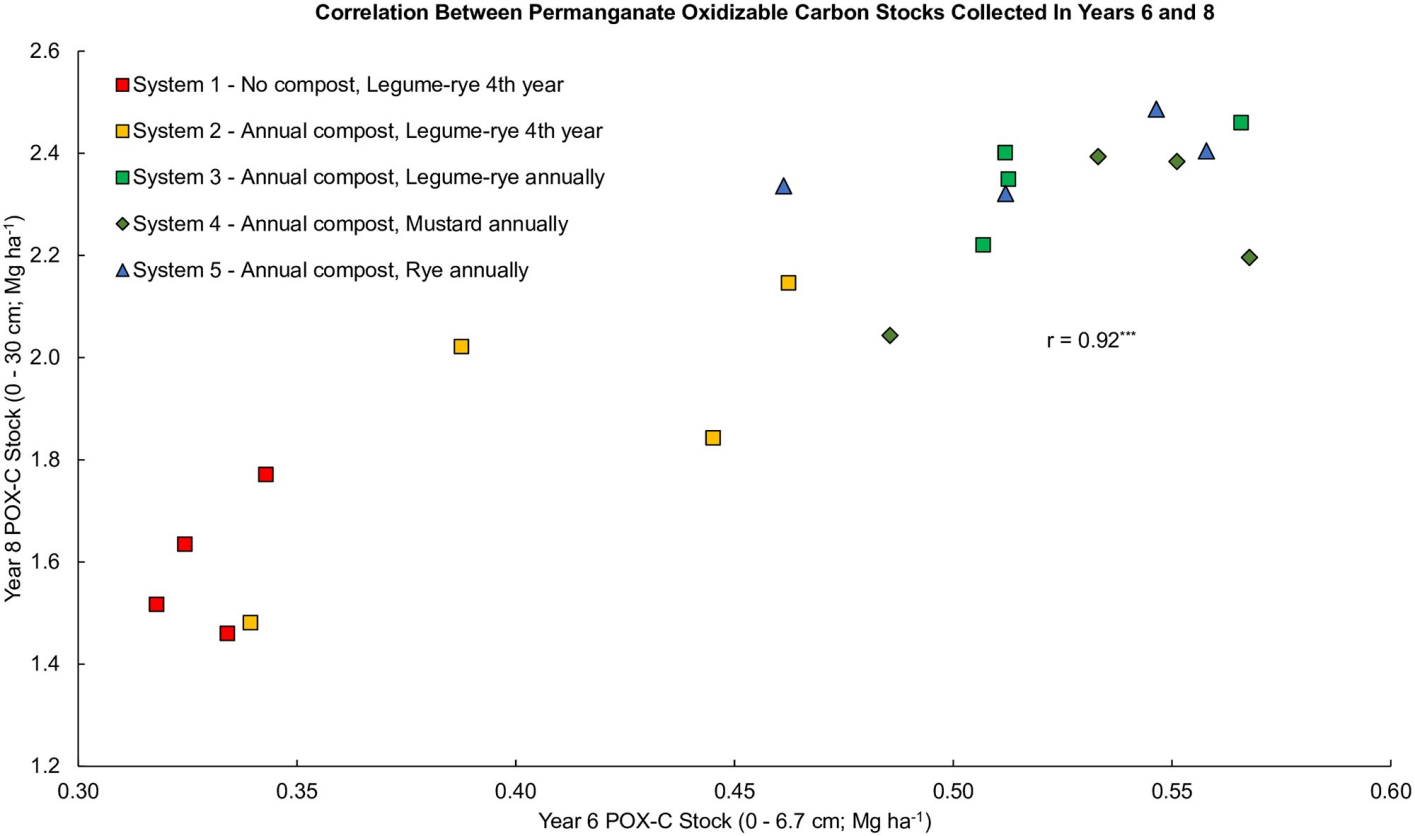
Correlation between permanganate oxidizable carbon stocks collected in years 6 and 8. Measurements were taken at the 0 to 6.7 cm depth in year 6 and at the 0 to 30 cm depth in year 8 in five organic vegetable systems in Salinas, CA. Systems differed by annual compost additions (0 vs. 7.6 Mg ha^-1^ before each vegetable crop), cover crop type (legume-rye, mustard or cereal rye alone) and cover cropping frequency (quadrennially vs. annually planted).

Measurements of POX-C were highly correlated between years 6 and 8 despite differences in sampling depth (Fig 9), reflecting the ongoing balance that had developed between cover crop C inputs (Fig 2) and POX-C over time. Though Fig 7A and 7B present different amounts of POX-C reflecting the different sampling depths, we calculated the linear relationship between the year 6 and year 8 POX-C data in Fig 9 and used that equation to determine the y-axis minima and maxima in Fig 7B. Thus, we present Fig 7A and 7B on an equivalent scale for easier comparison of the relative magnitude of treatment response. Visual comparison of these two figures reveals that the treatment effects on POX-C observed in year 6 were likewise evident in year 8 with comparable differences between the annually and quadrennially cover cropped systems. In contrast to the 0 to 6.7 cm depth in year 6, only increased cover crop frequency had a consistent positive effect on POX-C at the deeper sampling depth in year 8 (Fig 8B). Like year 6, POX-C in the annual legume-rye cover cropped System 3 was 48% greater than System 1 and 26% greater than System 2 in year 8. Similarly the mean for all the annually cover cropped systems was 46% greater than in System 1 and 25% greater than in System 2 (Fig 7B).

**Fig 10:**
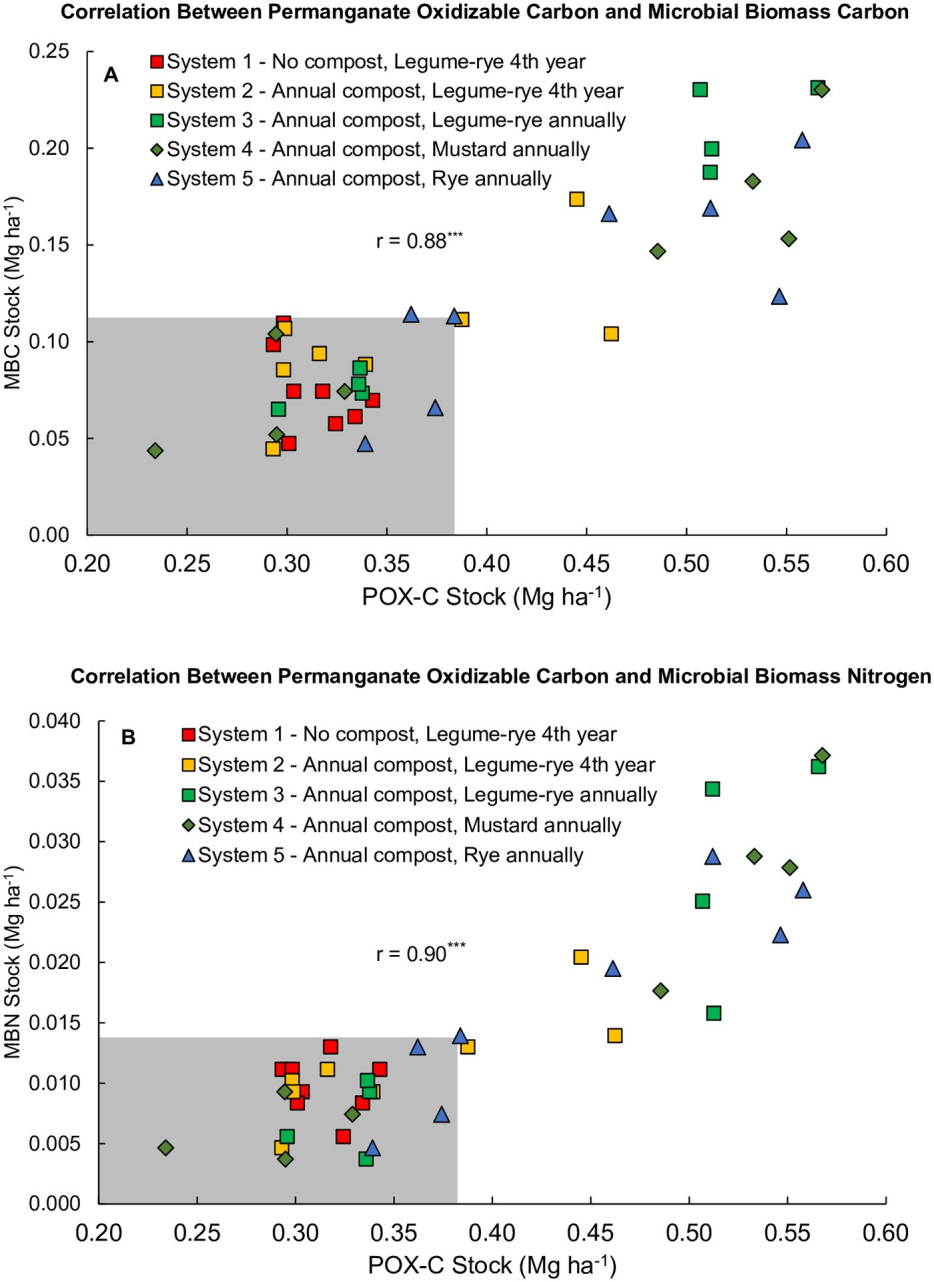
Correlation between permanganate oxidizable carbon and microbial biomass carbon (A) and microbial biomass nitrogen (B). Measurements were taken at the 0 to 6.7 cm depth in both years 0 and 6 in five organic vegetable systems in Salinas, CA. Systems differed by annual compost additions (0 vs. 7.6 Mg ha^-1^ before each vegetable crop), cover crop type (legume-rye, mustard or cereal rye alone) and cover cropping frequency (quadrennially vs. annually planted). There are eight raw data points for each system, including four for time 0 and four for year 6. The areas shaded grey contains the range of all data in year 0.

Numerous studies have demonstrated that the labile C fraction of SOC measured by POX-C is sensitive to management changes [68–71] and may be a useful tool for predicting cash crop response to improved SOC management [72]. Labile C is important for soil aggregate stabilization and C and N mineralization in soils [56,73]. As such, increasing labile C in organic vegetable production systems should help mitigate the deleterious effects of intensive tillage on soil structure and increase nutrient availability and cycling. Significant relationships have been found between POX-C and multiple measures of soil microbial activity such as microbial biomass C (MBC), substrate induced respiration and soluble carbohydrates [42,43]. Accordingly, the treatment effects on POX-C at the 0 to 6.7 cm depth in our study are similar to those reported for MBC, MBN and several soil enzymes on the same samples [18,31,32]. We likewise found strong positive correlations between our POX-C measurements and the MBC and MBN data previously reported by Brennan and Acosta Martinez [31] (Fig 10A and 10B). In addition, positive linear relationships have been demonstrated between POX-C and particulate organic C (POC) [43]. Culman et al.[43] demonstrated that POX-C is correlated with smaller and heavier POC fractions and thus measures a processed pool of labile SOC that is sensitive to differences in cropping system management.

Relatively few studies have assessed the direct effect of cover cropping on POX-C. In a Wisconsin silt loam, 4 years of clover, ryegrass and cereal rye cover crops interseeded in no-till silage corn increased POX-C to 15 cm depth, while liquid dairy manure amendment alone did not [74]. In Maryland, 12 years of cereal rye cover crops following continuous no-till corn increased POX-C to 7 cm in Coastal Plain silt loams, but not in finer textured Piedmont soils–possibly due to greater initial SOM in the latter soil [75]. Also in Maryland, Wang et al. [76] report that a single forage radish cover crop following no-till corn silage increased surface soil (0 to 15 cm) POX-C after the subsequent corn crop in Coastal Plain silt loams. In contrast, no effect on POX-C was observed due to a wheat-crimson clover winter cover crop after 2 years of eggplant and watermelon production on a twice annually tilled clay loam soil in Tennessee [77]. As the authors note, given that POX-C represents a processed labile SOC fraction, the short timeframe of the study may have been insufficient to observe a treatment effect. In comparison to the no-till studies, tillage in our study likely increased the mineralization rate of the cover crop derived labile C. Unlike the Tennessee study, the eight-year duration of our study allowed the treatment effect to become apparent despite the higher intensity of tillage.

## Conclusions

Multiple tillage and bed-forming operations per growing season, along with irrigation and fertilizer inputs can have a strong negative impact on SOC stocks in intensive vegetable production systems in the Salinas Valley. Yearly applications of urban yard waste compost (which, given the feedstocks, is presumably rich in lignin) helped to mitigate this effect by increasing the pool of resistant SOC. However, compost is an off-farm input that can contribute to excessive soil phosphorus if applied at rates greater than phosphorus removal in harvested crops, potentially impacting water quality and diminishing the sustainability of organic systems that ideally would be less reliant on off-farm inputs. Our study provides the first detailed information on C inputs from on-farm sources (i.e., cover crops and vegetable residue including estimates of below ground inputs) versus off-farm sources (compost, fertilizer, vegetable transplants) in tillage-intensive, high-input, organic vegetable production in California. It is important to highlight the need for more research on the below ground C inputs that will provide better estimates of shoot:root ratios of cash and cover crops in high-input systems. While this research occurred in an organic context, the results are also relevant to conventional vegetable farms in the region. Cover cropping increased C return to the soil directly following incorporation of cover crop shoots, and indirectly through higher vegetable biomass production and greater vegetable residue return to the soil that was likely due to improved nutrient cycling. It is important to note that we are not able to separate the effect of compost from increased cover crop frequency because the design of this study does not include annually cover cropped systems without compost application; this is an issue that should be evaluated in future research. Furthermore, our study only evaluated SOC stocks in the top 30 cm of the soil and may not reflect changes in subsoil SOC, which recently published research [20] suggests exerts a strong effect on overall SOC stocks throughout the soil profile. While compost application appears to have the greatest effect on total SOC stocks in our study, increasing cover crop frequency altered the composition of SOC by increasing the proportion of labile C, potentially increasing nutrient availability and thus likely having a greater impact on crop yields than compost. Our results also demonstrate the utility of POX-C as an inexpensive measure of the labile soil C fractions that may be most relevant to growers. To our knowledge this paper is the first to report the effect of cover cropping and intensive vegetable production management on POX-C in the Salinas Valley. Given the importance of labile C for soil health and the concomitant improvement in cropping system performance, these results emphasize the critical role of cover crops for maintaining this SOC fraction in frequently tilled and intensively managed soils.

## Supporting information

S1 Fig Cumulative organic matter inputs over eight years of vegetable production. Sum of mean organic matter inputs (oven dry basis) over eight years of intensive vegetable cropping in five organic vegetable systems in Salinas, CA. Inputs included pelleted organic fertilizer, vegetable transplant potting mix, urban yard waste compost, cover crops (roots and shoots) and vegetables (roots and post-harvest residue). Systems differed by annual compost additions (0 vs. 7.6 Mg ha^-1^before each vegetable crop), cover crop type (legume-rye, mustard, or cereal rye alone) and cover cropping frequency (quadrennially vs. annually planted). Inputs from cover crops include shoot and estimated root biomass, and for vegetables included post-harvest shoot biomass of broccoli and lettuce and estimates of their root biomass. Bar heights equal the sum of all organic matter inputs. Error bars are 95% confidence limits for total organic matter input. Mean total organic matter inputs (Mg ha^-1^) were 98.5 (System 1), 217.4 (System 2), 279.2 (System 3), 255.7 (System 4) and 271.4 (System 5). Total carbon means with the same letter above the x-axis to the right of each bar are not significantly different based on the Tukey-Kramer adjusted family-wise error rate of (P≤0.05).
(TIF)

S2 Fig Mean permanganate oxidizable carbon concentrations. Measurements were taken at the 0 to 6.7 cm depth for years 0 and 6 (A), the 0 to 30 cm depth in year 8 (B) in five organic vegetable systems in Salinas, CA. Systems differed by annual compost additions (0 vs. 7.6 Mg ha^-1^ before each vegetable crop), cover crop type (legume-rye, mustard, or cereal rye alone) and cover cropping frequency (quadrennially vs. annually planted). Error bars are 95% confidence limits. Individual data points for reps 1 through 4 of each system are clustered in order from left to right around the mean, which is represented by the horizontal lines.
(TIF)

S3 Fig Cover crop type effect on soil organic carbon stocks. Soil organic carbon stocks over 8 years in three organic vegetable systems in Salinas, CA that all received annual compost additions (7.6 Mg ha^-1^ before each vegetable crop) and differed by annually planted cover crop type.
(TIF)

S1 Table Summary statistics (means, 95% confidence limits, and standard errors) for carbon inputs, soil carbon stocks measured in years 0 through 8 and permanganate oxidizable carbon in years 0, 6 and 8. (DOCX)
