# Correction: Winter cover crops increase readily decomposable soil carbon, but compost drives total soil carbon during eight years of intensive, organic vegetable production in California

**DOI:** 10.1371/journal.pone.0307250

**Published:** 2024-07-11

**Authors:** Kathryn E. White, Eric B. Brennan, Michel A. Cavigelli, Richard F. Smith

In Figs 5–10, Table 2, S3 Fig and S1 Table, the carbon stocks are incorrect. Please see the correct Figs 5–10, Table 2, S3 Fig and S1 Table here.

**Fig 5:**
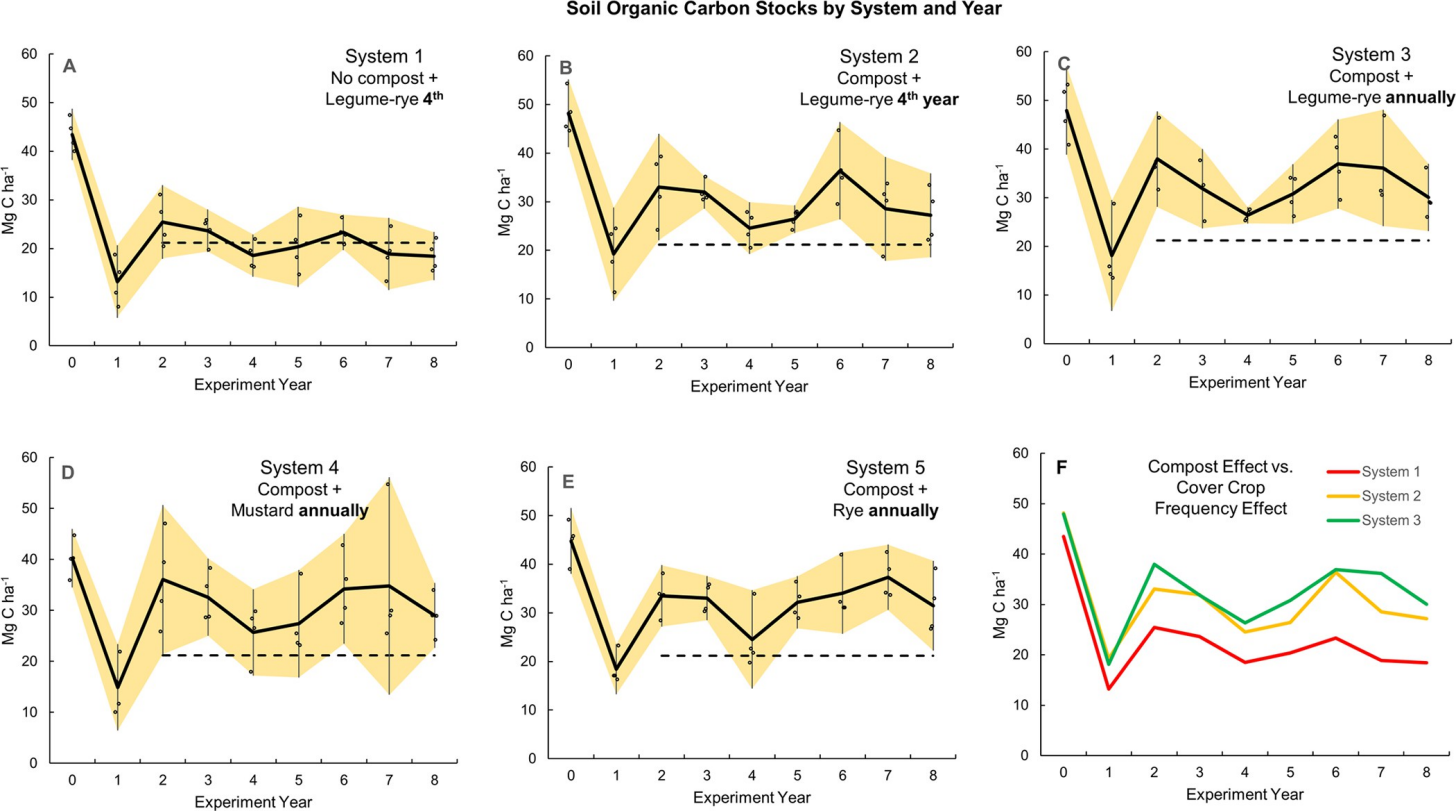
Soil organic carbon stocks by system and year. Measurements were taken at the 0 to 30 cm depth over 8 years in five organic vegetable systems in Salinas, CA. Systems differed by annual compost additions (0 vs. 7.6 Mg ha^-1^ before each vegetable crop), cover crop type (legume-rye, mustard, or cereal rye alone) and cover cropping frequency (quadrennially vs. annually planted). The dashed line indicates mean soil organic carbon between years 2 to 8 for System 1 and is included on each graph as a reference. Error bars are 95% confidence limits that are connected from year to year by the yellow band. Individual data points for reps 1 through 4 of each system are clustered around the mean.

**Fig 6:**
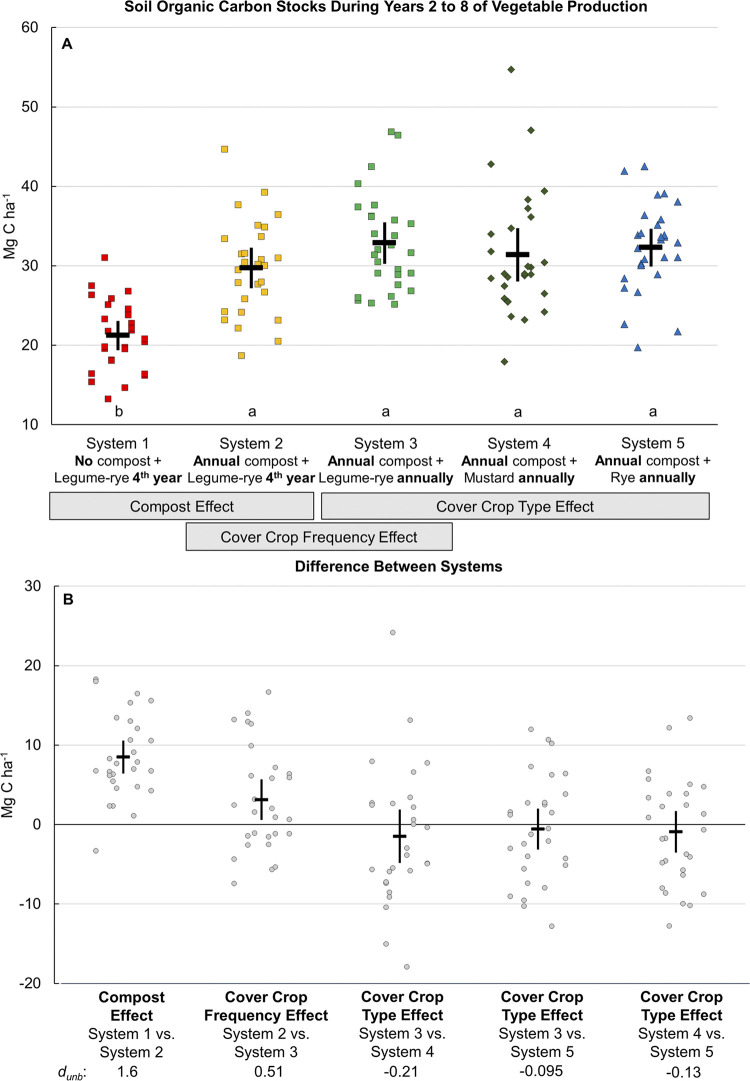
Soil organic carbon stocks during years 2 to 8 of vegetable production. Measurements were taken at the 0 to 30 cm depth. Mean soil organic carbon stocks for years 2 through 8 (A) and mean differences between systems (B) in five organic vegetable systems in Salinas, CA. Error bars are 95% confidence limits. Systems differed by annual compost additions (0 vs. 7.6 Mg ha^-1^ before each vegetable crop), cover crop type (legume-rye, mustard, or cereal rye alone) and cover cropping frequency (quadrennially vs. annually planted). In plot A the individual data points are in order of year 2 to 8 and replicate 1 to 4 from left to right; the same order is used in the difference between system pairs. The error bar in the center of the data cluster is the 95% confidence interval with the mean at the horizontal line. Means with the same letter above the x-axis in plot A are not significantly different based on the Tukey-Kramer adjusted family-wise error rate of (P≤0.05). The standardized effect size (Cohen’s unbiased *d*, *d*_unb_) is shown below the x-axis labels in plot B.

**Fig 7:**
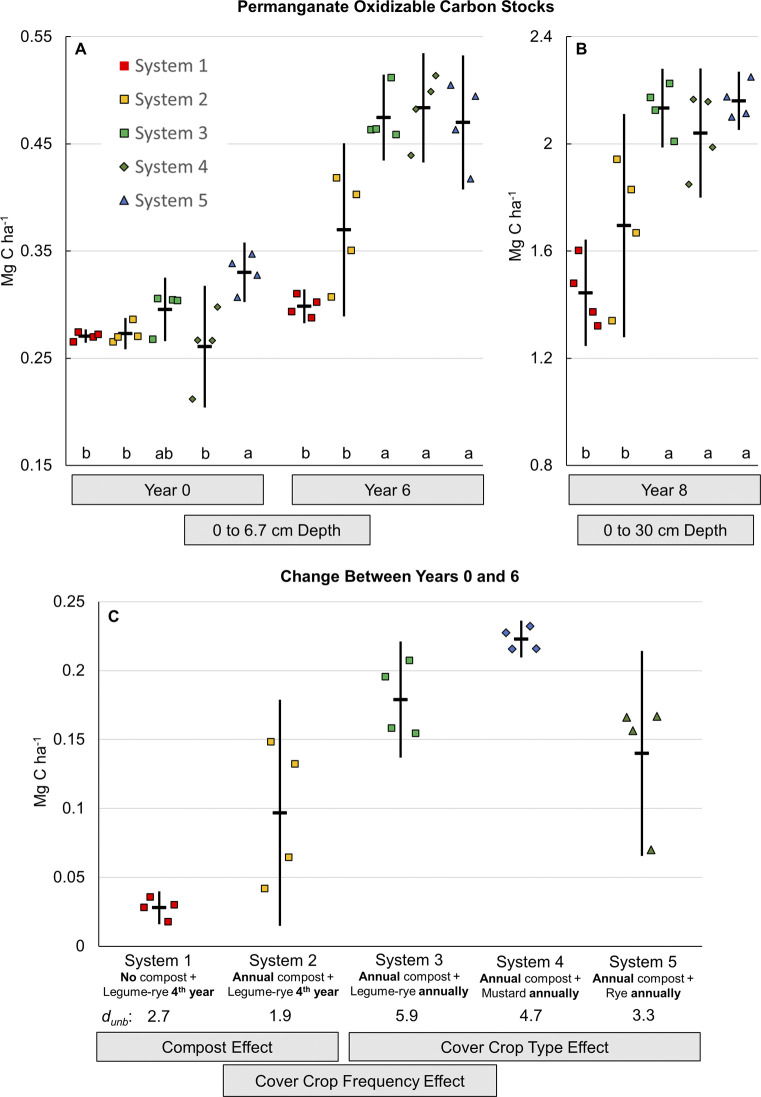
Mean permanganate oxidizable carbon stocks. Measurements were taken at the 0 to 6.7 cm depth for years 0 and 6 (A), the 0 to 30 cm depth in year 8 (B) and the change between year 0 and 6 at the 0 to 6.7 cm depth (C) in five organic vegetable systems in Salinas, CA. Systems differed by annual compost additions (0 vs. 7.6 Mg ha^-1^ before each vegetable crop), cover crop type (legume-rye, mustard, or cereal rye alone) and cover cropping frequency (quadrennially vs. annually planted). Error bars are 95% confidence limits. Individual data points for reps 1 through 4 of each system are clustered in order from left to right around the mean, which is represented by the horizontal lines. Mean permanganate oxidizable carbon stock at the 0 to 6.7 cm depth (Mg ha^-1^) was 24 (System 1), 33 (System 2), 36 (System 3), 35 (System 4) and 36 (System 5). Within a year, means with the same letter above the x-axis in plot A are not significantly different based on the Tukey-Kramer adjusted family-wise error rate of (P≤0.05). The standardized effect size (Cohen’s unbiased *d*, *d*_unb_) is shown below the x-axis labels in plot B.

**Fig 8:**
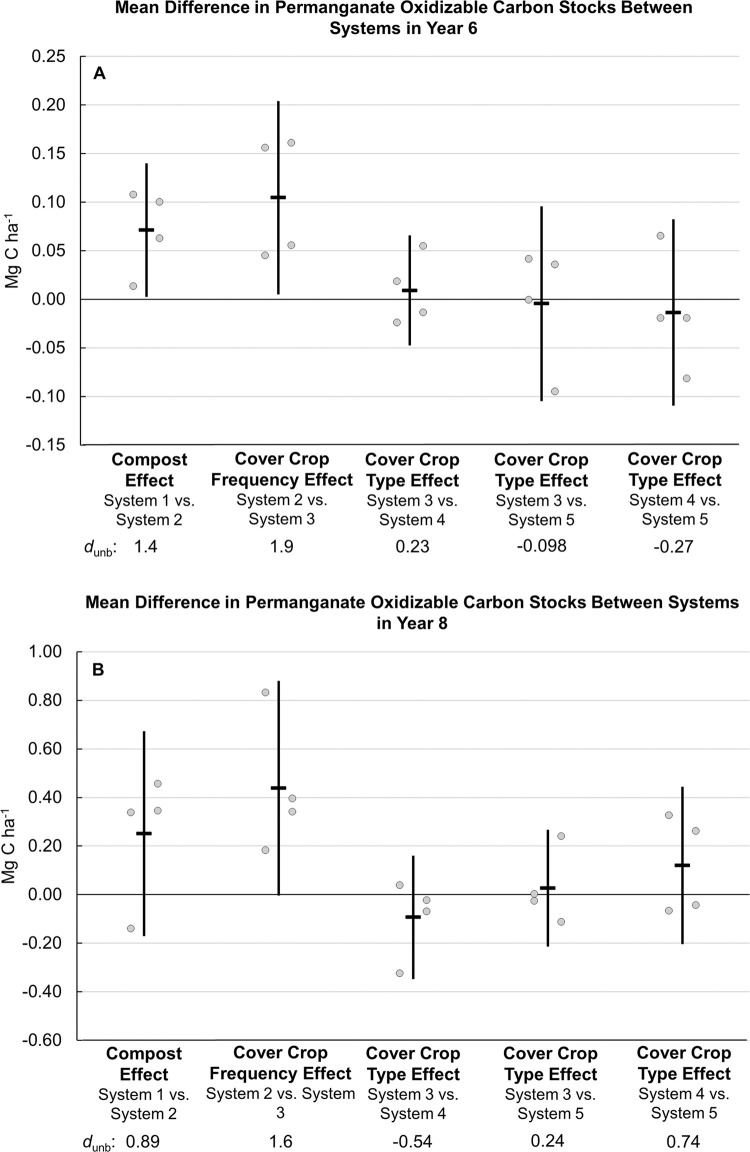
Mean difference in permanganate oxidizable carbon stocks between systems in years 6 and 8. Measurements were taken at the 0 to 6.7 cm depth for years 0 and 6 (A), and the 0 to 30 cm depth in year 8 (B). Systems differed by annual compost additions (0 vs. 7.6 Mg ha^-1^ before each vegetable crop), cover crop type (legume-rye, mustard, or cereal rye alone) and cover cropping frequency (quadrennially vs. annually planted). Error bars are 95% confidence limits. Individual data points for replicates 1 through 4 of each system are clustered in order from left to right around the mean, which is represented by the horizontal lines. The standardized effect size (Cohen’s unbiased *d*, *d*_unb_) is shown below the x-axis labels.

**Fig 9:**
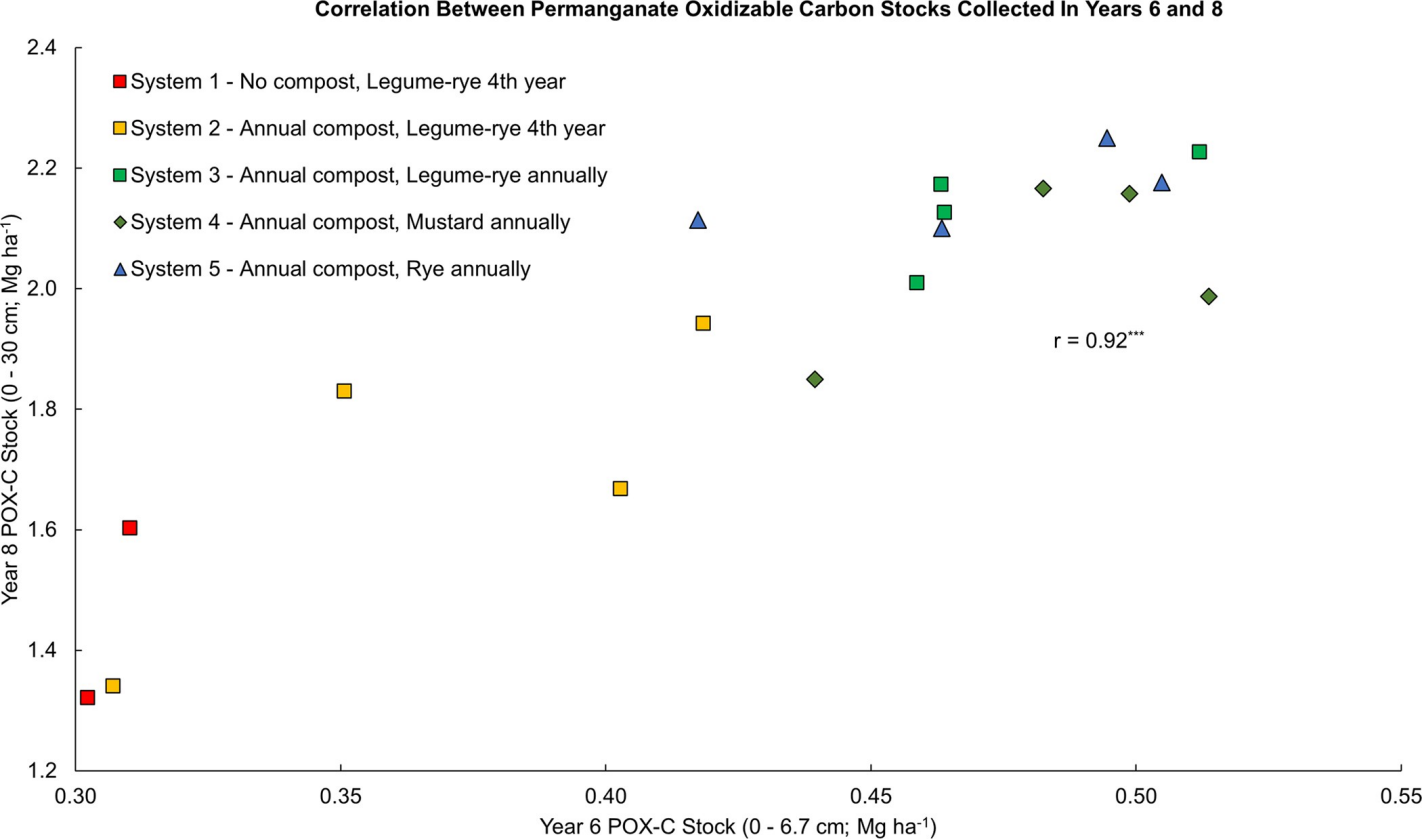
Correlation between permanganate oxidizable carbon stocks collected in years 6 and 8. Measurements were taken at the 0 to 6.7 cm depth in year 6 and at the 0 to 30 cm depth in year 8 in five organic vegetable systems in Salinas, CA. Systems differed by annual compost additions (0 vs. 7.6 Mg ha^-1^ before each vegetable crop), cover crop type (legume-rye, mustard or cereal rye alone) and cover cropping frequency (quadrennially vs. annually planted).

**Fig 10:**
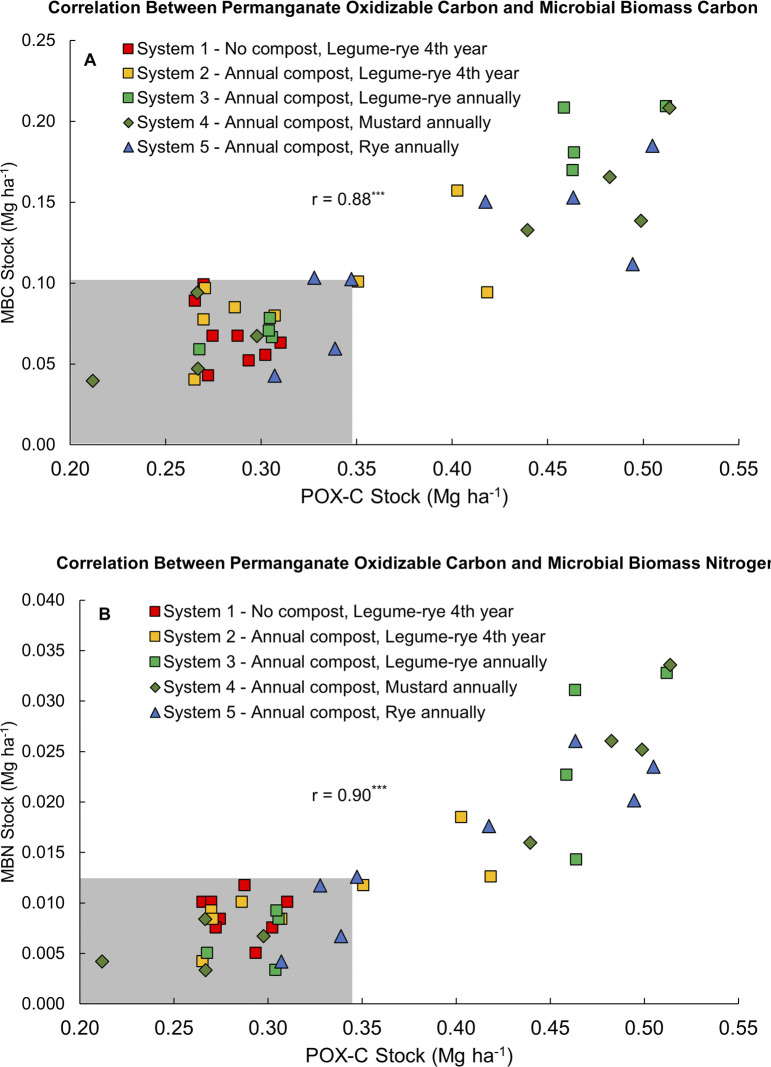
Correlation between permanganate oxidizable carbon and microbial biomass carbon (A) and microbial biomass nitrogen (B). Measurements were taken at the 0 to 6.7 cm depth in both years 0 and 6 in five organic vegetable systems in Salinas, CA. Systems differed by annual compost additions (0 vs. 7.6 Mg ha^-1^ before each vegetable crop), cover crop type (legume-rye, mustard or cereal rye alone) and cover cropping frequency (quadrennially vs. annually planted). There are eight raw data points for each system, including four for time 0 and four for year 6. The areas shaded grey contains the range of all data in year 0.

**Table 2:** Carbon inputs, soil organic carbon and permanganate oxidizable carbon ANOVA F-statistics and significance.

Effect	Total C Inputs		Cover Crop Shoot C Inputs		Vegetable Residue Shoot C Inputs		Soil Organic C		Permanganate Oxidizable C (0 to 6.7 cm)		Permanganate Oxidizable C (0 to 30 cm)	
System	2572	***	788.4	***	13.4	***	16.4	***	21.7	***	17.7	***
Year							10.8	***	308.5	***		
System*Year							0.60	n.s.	19.6	***		

*** P<0.001

## Supporting information

S3 Fig Cover crop type effect on soil organic carbon stocks. Soil organic carbon stocks over 8 years in three organic vegetable systems in Salinas, CA that all received annual compost additions (7.6 Mg ha-1 before each vegetable crop) and differed by annually planted cover crop type. (TIF)

S1 Table Summary statistics (means, 95% confidence limits, and standard errors) for carbon inputs, soil carbon stocks measured in years 0 through 8 and permanganate oxidizable carbon in years 0, 6 and 8. (DOCX)
