# Supplementary figures and images for: Winter cover crops increase readily decomposable soil carbon, but compost drives total soil carbon during eight years of intensive, organic vegetable production in California
Source: PLoS One. 2020 Feb 6;15(2):e0228677. doi: 10.1371/journal.pone.0228677 (PMC7004306; doi:10.1371/journal.pone.0228677)

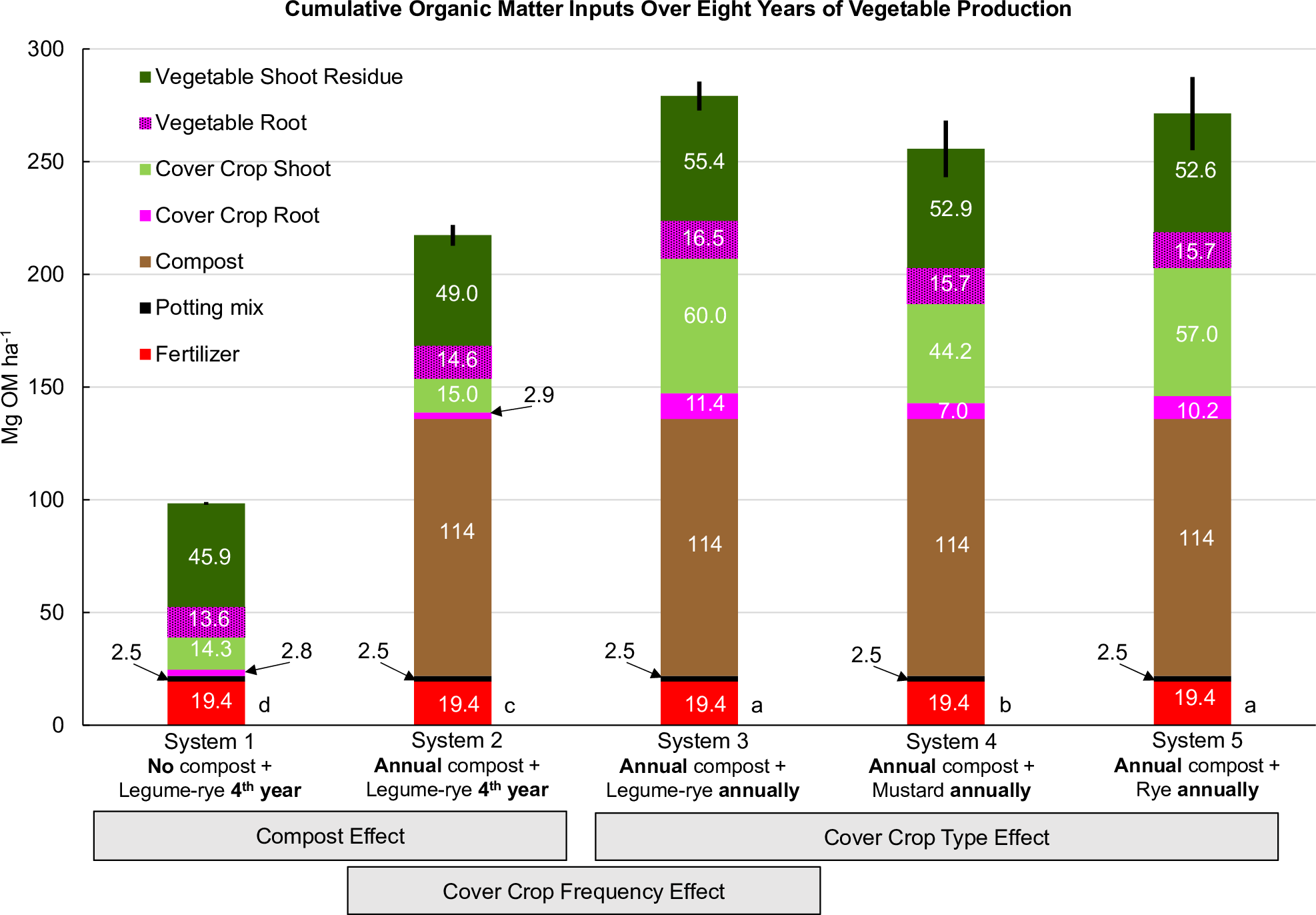

Supplement: S1 Fig — Sum of mean organic matter inputs (oven dry basis) over eight years of intensive vegetable cropping in five organic vegetable systems in Salinas, CA. Inputs included pelleted organic fertilizer, vegetable transplant potting mix, urban yard waste compost, cover crops (roots and shoots) and vegetables (roots and post-harvest residue). Systems differed by annual compost additions (0 vs. 7.6 Mg ha-1before each vegetable crop), cover crop type (legume-rye, mustard, or cereal rye alone) and cover cropping frequency (quadrennially vs. annually planted). Inputs from cover crops include shoot and estimated root biomass, and for vegetables included post-harvest shoot biomass of broccoli and lettuce and estimates of their root biomass. Bar heights equal the sum of all organic matter inputs. Error bars are 95% confidence limits for total organic matter input. Mean total organic matter inputs (Mg ha-1) were 98.5 (System 1), 217.4 (System 2), 279.2 (System 3), 255.7 (System 4) and 271.4 (System 5). Total carbon means with the same letter above the x-axis to the right of each bar are not significantly different based on the Tukey-Kramer adjusted family-wise error rate of (P≤0.05). (TIF) [file pone.0228677.s001.tif]

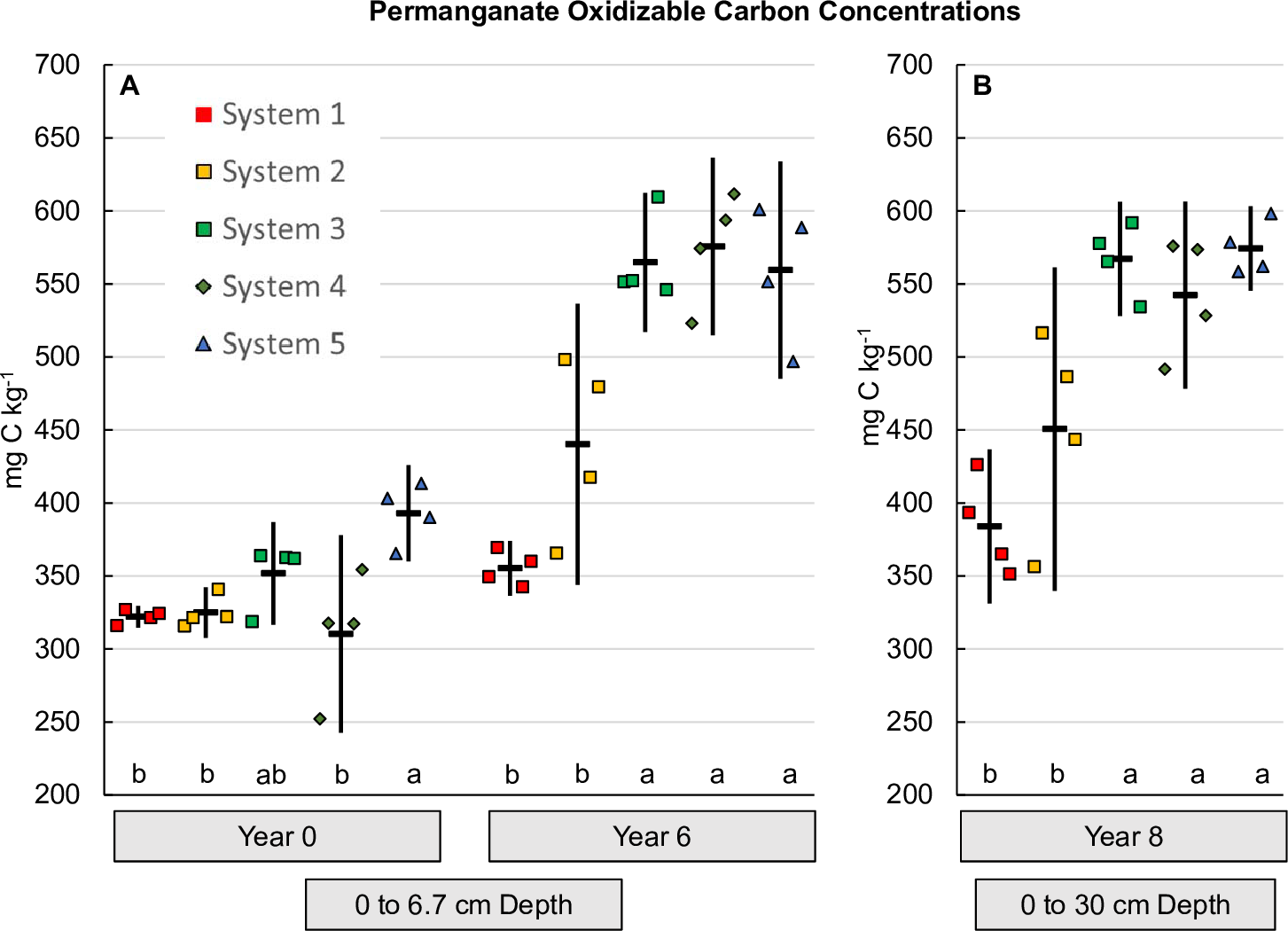

Supplement: S2 Fig — Measurements were taken at the 0 to 6.7 cm depth for years 0 and 6 (A), the 0 to 30 cm depth in year 8 (B) in five organic vegetable systems in Salinas, CA. Systems differed by annual compost additions (0 vs. 7.6 Mg ha-1 before each vegetable crop), cover crop type (legume-rye, mustard, or cereal rye alone) and cover cropping frequency (quadrennially vs. annually planted). Error bars are 95% confidence limits. Individual data points for reps 1 through 4 of each system are clustered in order from left to right around the mean, which is represented by the horizontal lines. (TIF) [file pone.0228677.s002.tif]

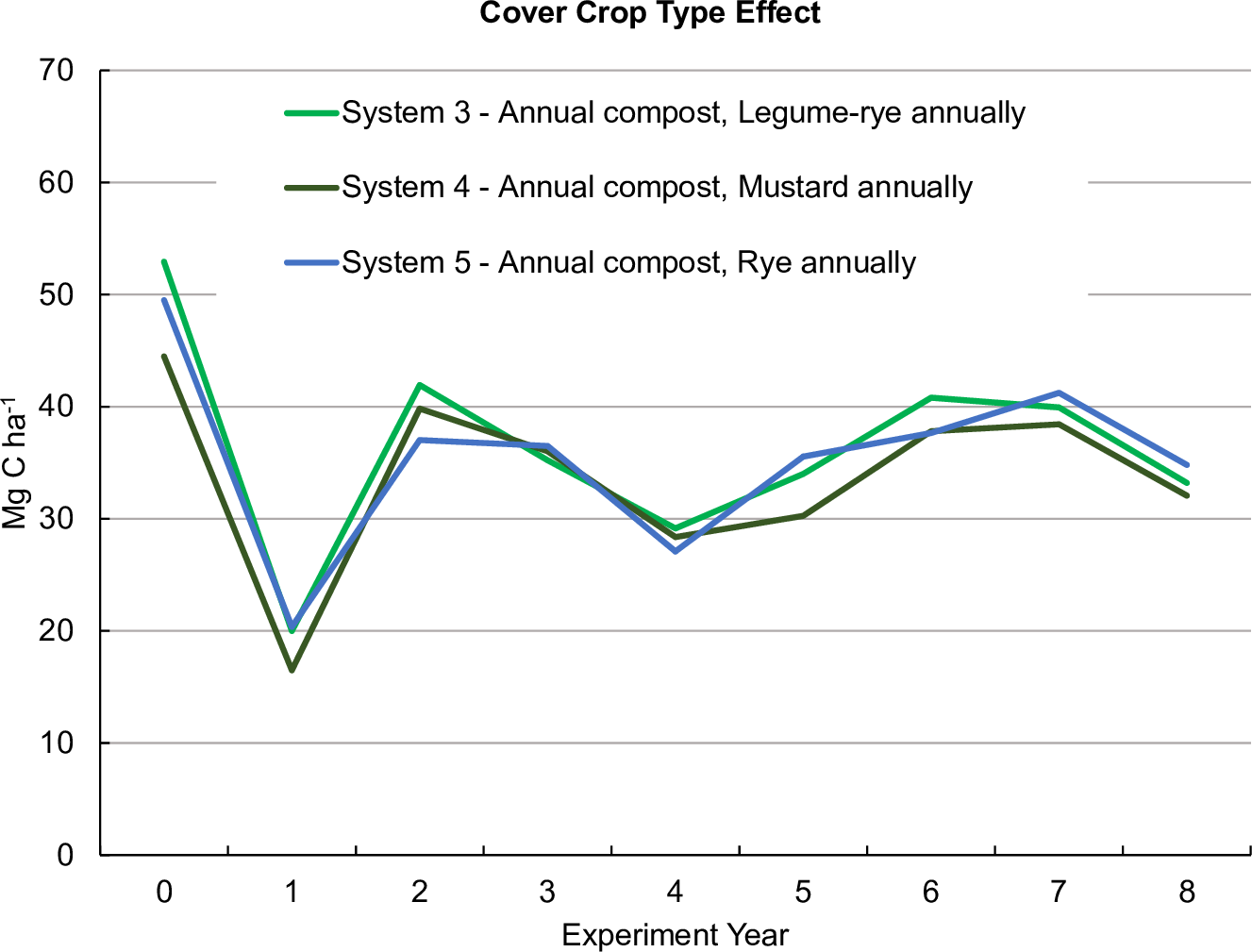

Supplement: S3 Fig — Soil organic carbon stocks over 8 years in three organic vegetable systems in Salinas, CA that all received annual compost additions (7.6 Mg ha-1 before each vegetable crop) and differed by annually planted cover crop type. (TIF) [file pone.0228677.s003.tif]
