# Supplementary material for: Winter cover crops increase readily decomposable soil carbon, but compost drives total soil carbon during eight years of intensive, organic vegetable production in California
Source: PLoS One. 2020 Feb 6;15(2):e0228677. doi: 10.1371/journal.pone.0228677 (PMC7004306; doi:10.1371/journal.pone.0228677)
Supplement: S1 Table — (DOCX) [file pone.0228677.s004.docx]

S1 Table. Summary statistics (means, 95% confidence limits, and standard errors) for carbon inputs, soil carbon stocks measured in years 0 through 8 and permanganate oxidizable carbon in years 0, 6 and 8.

|  |  | System 1 | System 2 | System 3 | System 4 | System 5 |
| --- | --- | --- | --- | --- | --- | --- |
|  |  | Mg C ha^-1^ | | | | |
| Vegetable Shoot Residue C | Mean | 16.9 | 18.0 | 20.4 | 19.5 | 19.4 |
|  | Confidence Limits | 15.9, 17.9 | 16.7, 19.3 | 19.3, 21.5 | 17.5, 21.4 | 17.3, 21.4 |
|  | Standard Error | 0.31 | 0.41 | 0.35 | 0.62 | 0.64 |
|  |  |  |  |  |  |  |
| Vegetable Root C | Mean | 5.0 | 5.4 | 6.1 | 5.8 | 5.8 |
|  | Confidence Limits | 4.7, 5.3 | 5.0, 5.7 | 5.8, 6.4 | 5.2, 6.4 | 5.2, 6.4 |
|  | Standard Error | 0.092 | 0.12 | 0.10 | 0.18 | 0.18 |
|  |  |  |  |  |  |  |
| Vegetable Root Exudate C | Mean | 3.3 | 3.5 | 4.0 | 3.8 | 3.8 |
|  | Confidence Limits | 3.1, 3.4 | 3.2, 3.7 | 3.7, 4.2 | 3.4, 4.1 | 3.4, 4.1 |
|  | Standard Error | 0.06 | 0.08 | 0.06 | 0.11 | 0.12 |
|  |  |  |  |  |  |  |
| Cover Crop Shoot C | Mean | 6.3 | 6.6 | 26.4 | 18.6 | 25.2 |
|  | Confidence Limits | 5.2, 7.4 | 6.2, 7.1 | 24.9, 27.9 | 16.1, 21.1 | 21.4, 28.9 |
|  | Standard Error | 0.34 | 0.14 | 0.47 | 0.77 | 1.18 |
|  |  |  |  |  |  |  |
| Cover Crop Root C | Mean | 1.2 | 1.3 | 5.0 | 3.0 | 4.5 |
|  | Confidence Limits | 1.0, 1.4 | 1.2, 1.4 | 4.7, 5.3 | 2.6, 3.3 | 3.8, 5.2 |
|  | Standard Error | 0.06 | 0.02 | 0.10 | 0.12 | 0.21 |
|  |  |  |  |  |  |  |
| Cover Crop Root Exudate C | Mean | 0.79 | 0.83 | 3.3 | 1.9 | 2.9 |
|  | Confidence Limits | 0.7, 0.9 | 0.8, 0.9 | 3.1, 3.5 | 1.7, 2.2 | 2.5, 3.4 |
|  | Standard Error | 0.04 | 0.02 | 0.07 | 0.08 | 0.14 |
|  |  |  |  |  |  |  |
| Soil C Stock, Year 0 | Mean | 48.0 | 53.2 | 52.9 | 44.5 | 49.5 |
|  | Confidence Limits | 42.2, 53.8 | 45.5, 60.9 | 42.9, 62.9 | 38.2, 50.8 | 42.1, 56.9 |
|  | Standard Error | 1.83 | 2.42 | 3.14 | 1.99 | 2.34 |
|  |  |  |  |  |  |  |
| Soil C Stock, Year 1 | Mean | 14.6 | 21.2 | 20.0 | 16.5 | 20.4 |
|  | Confidence Limits | 6.3, 22.8 | 10.6, 31.8 | 7.4, 32.6 | 7.1, 25.8 | 14.7, 26.0 |
|  | Standard Error | 2.60 | 3.33 | 3.96 | 2.94 | 1.79 |
|  |  |  |  |  |  |  |
| Soil C Stock, Year 2 | Mean | 28.1 | 36.5 | 41.9 | 39.8 | 37.0 |
|  | Confidence Limits | 19.7, 36.5 | 24.4, 48.6 | 31.1, 52.8 | 23.6, 56.0 | 30.1, 44.0 |
|  | Standard Error | 2.63 | 3.80 | 3.42 | 5.08 | 2.19 |
|  |  |  |  |  |  |  |
| Soil C Stock, Year 3 | Mean | 26.1 | 35.3 | 35.2 | 36.0 | 36.5 |
|  | Confidence Limits | 21.3, 30.9 | 31.6, 39.1 | 26.2, 44.2 | 27.7, 44.4 | 31.5, 41.5 |
|  | Standard Error | 1.51 | 1.18 | 2.84 | 2.63 | 1.58 |
|  |  |  |  |  |  |  |
| Soil C Stock, Year 4 | Mean | 20.5 | 27.1 | 29.1 | 28.4 | 27.1 |
|  | Confidence Limits | 15.7, 25.3 | 21.2, 33.1 | 27.3, 31.0 | 19.0, 37.7 | 15.9, 38.3 |
|  | Standard Error | 1.51 | 1.86 | 0.59 | 2.94 | 3.52 |
|  |  |  |  |  |  |  |
| Soil C Stock, Year 5 | Mean | 22.5 | 29.2 | 34.0 | 30.3 | 35.5 |
|  | Confidence Limits | 13.4, 31.6 | 26.1, 32.3 | 37.3, 40.7 | 18.6, 41.9 | 29.6, 41.5 |
|  | Standard Error | 2.87 | 0.98 | 2.12 | 3.66 | 1.86 |
|  |  |  |  |  |  |  |
| Soil C Stock, Year 6 | Mean | 25.8 | 40.2 | 40.8 | 37.8 | 37.7 |
|  | Confidence Limits | 21.7, 29.8 | 29.2, 51.2 | 30.7, 50.9 | 25.9, 49.7 | 28.4, 46.9 |
|  | Standard Error | 1.27 | 3.47 | 3.18 | 3.74 | 2.91 |
|  |  |  |  |  |  |  |
| Soil C Stock, Year 7 | Mean | 20.8 | 31.5 | 39.9 | 38.4 | 41.2 |
|  | Confidence Limits | 12.6, 29.0 | 19.7, 43.3 | 26.7, 53.2 | 48.8, 62.0 | 33.8, 48.6 |
|  | Standard Error | 2.58 | 3.71 | 4.16 | 7.41 | 2.33 |
|  |  |  |  |  |  |  |
| Soil C Stock, Year 8 | Mean | 20.4 | 30.1 | 33.2 | 32.1 | 34.8 |
|  | Confidence Limits | 14.9, 25.6 | 20.5, 39.6 | 25.6, 40.8 | 25.0, 39.1 | 24.6, 45.0 |
|  | Standard Error | 1.61 | 2.98 | 2.37 | 2.17 | 3.14 |
|  |  |  |  |  |  |  |
| POX-C Stock, Year 0 | Mean | 0.30 | 0.30 | 0.33 | 0.29 | 0.36 |
| (0 to 6.7 cm depth) | Confidence Limits | 0.29, 0.31 | 0.29, 0.32 | 0.29, 0.36 | 0.23, 0.35 | 0.33, 0.40 |
|  | Standard Error | 0.002 | 0.005 | 0.010 | 0.020 | 0.010 |
|  |  |  |  |  |  |  |
| POX-C Stock, Year 6 | Mean | 0.33 | 0.41 | 0.52 | 0.53 | 0.52 |
| (0 to 6.7 cm depth) | Confidence Limits | 0.31, 0.35 | 0.32, 0.50 | 0.48, 0.57 | 0.48, 0.59 | 0.45, 0.59 |
|  | Standard Error | 0.005 | 0.028 | 0.014 | 0.018 | 0.022 |
|  |  |  |  |  |  |  |
| POX-C Stock, Year 8 | Mean | 1.6 | 1.9 | 2.4 | 2.3 | 2.4 |
| (0 to 30 cm depth) | Confidence Limits | 1.4, 1.8 | 1.4, 2.3 | 2.2, 2.5 | 2.0, 2.5 | 2.3, 2.5 |
|  | Standard Error | 0.07 | 0.14 | 0.05 | 0.08 | 0.04 |
