# Supplementary figures and images for: Correction: Winter cover crops increase readily decomposable soil carbon, but compost drives total soil carbon during eight years of intensive, organic vegetable production in California
Source: PLoS One. 2024 Jul 11;19(7):e0307250. doi: 10.1371/journal.pone.0307250 (PMC11239057; doi:10.1371/journal.pone.0307250)

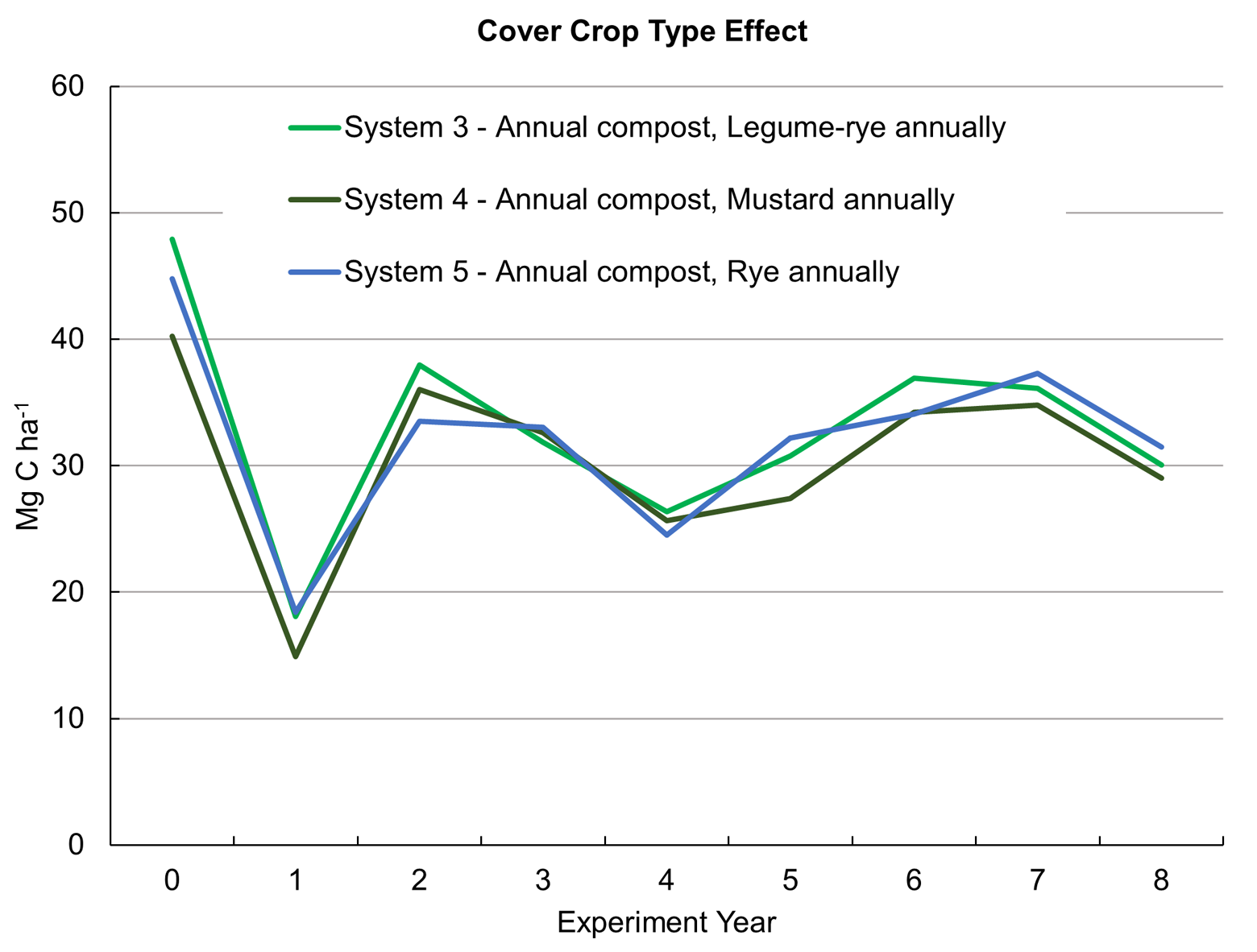

Supplement: S3 Fig — (TIF) [file pone.0307250.s001.tif]
