# Supplementary material for: Correction: Winter cover crops increase readily decomposable soil carbon, but compost drives total soil carbon during eight years of intensive, organic vegetable production in California
Source: PLoS One. 2024 Jul 11;19(7):e0307250. doi: 10.1371/journal.pone.0307250 (PMC11239057; doi:10.1371/journal.pone.0307250)
Supplement: S1 Table — (DOCX) [file pone.0307250.s002.docx]

S1 Table. Summary statistics (means, 95% confidence limits, and standard errors) for carbon inputs, soil carbon stocks measured in years 0 through 8 and permanganate oxidizable carbon in years 0, 6 and 8.

|  |  | System 1 | System 2 | System 3 | System 4 | System 5 |
| --- | --- | --- | --- | --- | --- | --- |
|  |  | Mg C ha^-1^ | | | | |
| Vegetable Shoot Residue C | Mean | 16.9 | 18.0 | 20.4 | 19.5 | 19.4 |
|  | Confidence Limits | 15.9, 17.9 | 16.7, 19.3 | 19.3, 21.5 | 17.5, 21.4 | 17.3, 21.4 |
|  | Standard Error | 0.31 | 0.41 | 0.35 | 0.62 | 0.64 |
|  |  |  |  |  |  |  |
| Vegetable Root C | Mean | 5.0 | 5.4 | 6.1 | 5.8 | 5.8 |
|  | Confidence Limits | 4.7, 5.3 | 5.0, 5.7 | 5.8, 6.4 | 5.2, 6.4 | 5.2, 6.4 |
|  | Standard Error | 0.092 | 0.12 | 0.10 | 0.18 | 0.18 |
|  |  |  |  |  |  |  |
| Vegetable Root Exudate C | Mean | 3.3 | 3.5 | 4.0 | 3.8 | 3.8 |
|  | Confidence Limits | 3.1, 3.4 | 3.2, 3.7 | 3.7, 4.2 | 3.4, 4.1 | 3.4, 4.1 |
|  | Standard Error | 0.06 | 0.08 | 0.06 | 0.11 | 0.12 |
|  |  |  |  |  |  |  |
| Cover Crop Shoot C | Mean | 6.3 | 6.6 | 26.4 | 18.6 | 25.2 |
|  | Confidence Limits | 5.2, 7.4 | 6.2, 7.1 | 24.9, 27.9 | 16.1, 21.1 | 21.4, 28.9 |
|  | Standard Error | 0.34 | 0.14 | 0.47 | 0.77 | 1.18 |
|  |  |  |  |  |  |  |
| Cover Crop Root C | Mean | 1.2 | 1.3 | 5.0 | 3.0 | 4.5 |
|  | Confidence Limits | 1.0, 1.4 | 1.2, 1.4 | 4.7, 5.3 | 2.6, 3.3 | 3.8, 5.2 |
|  | Standard Error | 0.06 | 0.02 | 0.10 | 0.12 | 0.21 |
|  |  |  |  |  |  |  |
| Cover Crop Root Exudate C | Mean | 0.79 | 0.83 | 3.3 | 1.9 | 2.9 |
|  | Confidence Limits | 0.7, 0.9 | 0.8, 0.9 | 3.1, 3.5 | 1.7, 2.2 | 2.5, 3.4 |
|  | Standard Error | 0.04 | 0.02 | 0.07 | 0.08 | 0.14 |
|  |  |  |  |  |  |  |
| Soil C Stock, Year 0 | Mean | 43.5 | 48.2 | 47.9 | 40.2 | 44.8 |
|  | Confidence Limits | 38.2, 48.7 | 41.2, 55.2 | 38.8, 57 | 34.5, 46 | 38.1, 51.5 |
|  | Standard Error | 1.66 | 2.19 | 2.85 | 1.80 | 2.12 |
|  |  |  |  |  |  |  |
| Soil C Stock, Year 1 | Mean | 13.2 | 19.2 | 18.1 | 14.9 | 18.4 |
|  | Confidence Limits | 5.7, 20.7 | 9.6, 28.8 | 6.7, 29.5 | 6.4, 23.4 | 13.3, 23.6 |
|  | Standard Error | 2.35 | 3.01 | 3.59 | 2.66 | 1.62 |
|  |  |  |  |  |  |  |
| Soil C Stock, Year 2 | Mean | 25.4 | 33.0 | 38.0 | 36.0 | 33.5 |
|  | Confidence Limits | 17.9, 33 | 22.1, 44 | 28.1, 47.8 | 21.4, 50.7 | 27.2, 39.8 |
|  | Standard Error | 2.38 | 3.44 | 3.09 | 4.60 | 1.98 |
|  |  |  |  |  |  |  |
| Soil C Stock, Year 3 | Mean | 23.6 | 32.0 | 31.9 | 32.6 | 33.0 |
|  | Confidence Limits | 19.3, 28 | 28.6, 35.4 | 23.7, 40 | 25, 40.2 | 28.5, 37.6 |
|  | Standard Error | 1.37 | 1.06 | 2.57 | 2.38 | 1.43 |
|  |  |  |  |  |  |  |
| Soil C Stock, Year 4 | Mean | 18.5 | 24.6 | 26.4 | 25.7 | 24.5 |
|  | Confidence Limits | 14.2, 22.9 | 19.2, 29.9 | 24.7, 28.1 | 17.2, 34.2 | 14.4, 34.6 |
|  | Standard Error | 1.36 | 1.68 | 0.53 | 2.66 | 3.18 |
|  |  |  |  |  |  |  |
| Soil C Stock, Year 5 | Mean | 20.4 | 26.4 | 30.8 | 27.4 | 32.2 |
|  | Confidence Limits | 12.1, 28.6 | 23.6, 29.2 | 24.7, 36.9 | 16.8, 37.9 | 26.8, 37.5 |
|  | Standard Error | 2.59 | 0.89 | 1.92 | 3.31 | 1.69 |
|  |  |  |  |  |  |  |
| Soil C Stock, Year 6 | Mean | 23.3 | 36.4 | 36.9 | 34.2 | 34.1 |
|  | Confidence Limits | 19.7, 27 | 26.4, 46.4 | 27.7, 46.1 | 23.4, 45 | 25.7, 42.5 |
|  | Standard Error | 1.15 | 3.14 | 2.88 | 3.39 | 2.63 |
|  |  |  |  |  |  |  |
| Soil C Stock, Year 7 | Mean | 18.9 | 28.5 | 36.1 | 34.8 | 37.3 |
|  | Confidence Limits | 11.4, 26.3 | 17.8, 39.2 | 24.2, 48.1 | 13.4, 56.1 | 30.6, 44 |
|  | Standard Error | 2.33 | 3.36 | 3.76 | 6.71 | 2.11 |
|  |  |  |  |  |  |  |
| Soil C Stock, Year 8 | Mean | 18.4 | 27.2 | 30.0 | 29.0 | 31.5 |
|  | Confidence Limits | 13.5, 23.4 | 18.6, 35.8 | 23.1, 36.9 | 22.6, 35.4 | 22.3, 40.7 |
|  | Standard Error | 1.56 | 2.72 | 2.17 | 2.00 | 2.90 |
|  |  |  |  |  |  |  |
| POX-C Stock, Year 0 | Mean | 0.27 | 0.27 | 0.30 | 0.26 | 0.33 |
| (0 to 6.7 cm depth) | Confidence Limits | 0.27, 0.28 | 0.26, 0.29 | 0.27, 0.32 | 0.21, 0.31 | 0.31, 0.35 |
|  | Standard Error | 0.002 | 0.004 | 0.008 | 0.015 | 0.008 |
|  |  |  |  |  |  |  |
| POX-C Stock, Year 6 | Mean | 0.30 | 0.37 | 0.47 | 0.48 | 0.47 |
| (0 to 6.7 cm depth) | Confidence Limits | 0.28, 0.31 | 0.3, 0.44 | 0.44, 0.51 | 0.44, 0.53 | 0.42, 0.52 |
|  | Standard Error | 0.004 | 0.022 | 0.011 | 0.014 | 0.017 |
|  |  |  |  |  |  |  |
| POX-C Stock, Year 8 | Mean | 1.4 | 1.7 | 2.1 | 2.0 | 2.2 |
| (0 to 30 cm depth) | Confidence Limits | 1.3, 1.6 | 1.3, 2.1 | 2, 2.3 | 1.8, 2.2 | 2.1, 2.3 |
|  | Standard Error | 0.05 | 0.11 | 0.04 | 0.07 | 0.03 |
